# Effects of vegetation management intensity on biodiversity and ecosystem services in vineyards: A meta‐analysis

**DOI:** 10.1111/1365-2664.13124

**Published:** 2018-03-04

**Authors:** Silvia Winter, Thomas Bauer, Peter Strauss, Sophie Kratschmer, Daniel Paredes, Daniela Popescu, Blanca Landa, Gema Guzmán, José A. Gómez, Muriel Guernion, Johann G. Zaller, Péter Batáry

**Affiliations:** ^1^ Institute of Integrative Nature Conservation Research and Division of Plant Protection University of Natural Resources and Life Sciences Vienna Austria; ^2^ Institute for Land and Water Management Research Austrian Federal Agency for Water Management Petzenkirchen Austria; ^3^ Enviromental Protection Department Estación Experimental del Zaidín Spanish Council of Research Granada Spain; ^4^ Faculty of Horticulture University of Agricultural Sciences and Veterinary Medicine Cluj‐Napoca Cluj‐Napoca Romania; ^5^ Institute for Sustainable Agriculture CSIC Cordoba Spain; ^6^ Université de Rennes I OSUR UMR CNRS 6553 ‘EcoBio’ OSUR Paimpont France; ^7^ Institute of Zoology University of Natural Resources and Life Sciences Vienna Austria; ^8^ Agroecology University of Goettingen Göttingen Germany; ^9^ GINOP Sustainable Ecosystems Group MTA Centre for Ecological Research Tihany Hungary

**Keywords:** biodiversity, carbon sequestration, ecosystem services, meta‐analysis, pest control, soil erosion, tillage intensity, vineyard

## Abstract

1. At the global scale, vineyards are usually managed intensively to optimize wine production without considering possible negative impacts on biodiversity and ecosystem services (ES) such as high soil erosion rates, degradation of soil fertility or contamination of groundwater. Winegrowers regulate competition for water and nutrients between the vines and inter‐row vegetation by tilling, mulching and/or herbicide application. Strategies for more sustainable viticulture recommend maintaining vegetation cover in inter‐rows, however, there is a lack of knowledge as to what extent this less intensive inter‐row management affects biodiversity and associated ES.
2. We performed a hierarchical meta‐analysis to quantify the effects of extensive vineyard inter‐row vegetation management in comparison to more intensive management (like soil tillage or herbicide use) on biodiversity and ES from 74 studies covering four continents and 13 wine‐producing countries.
3. Overall, extensive vegetation management increased above‐ and below‐ground biodiversity and ecosystem service provision by 20% in comparison to intensive management. Organic management together with management without herbicides showed a stronger positive effect on ES and biodiversity provision than inter‐row soil tillage.
4. Soil loss parameters showed the largest positive response to inter‐row vegetation cover. The second highest positive response was observed for biodiversity variables, followed by carbon sequestration, pest control and soil fertility. We found no trade‐off between grape yield and quality vs. biodiversity or other ES.
5. *Synthesis and applications*. Our meta‐analysis concludes that vegetation cover in inter‐rows contributes to biodiversity conservation and provides multiple ecosystem services. However, in drier climates grape yield might decrease without irrigation and careful vegetation management. Agri‐environmental policies should therefore focus on granting subsidies for the establishment of locally adapted diverse vegetation cover in vineyard inter‐rows. Future studies should focus on analysing the combined effects of local vineyard management and landscape composition and advance research in wine‐growing regions in Asia and in the southern hemisphere.

## Abstract 2

Our meta‐analysis concludes that vegetation cover in inter‐rows contributes to biodiversity conservation and provides multiple ecosystem services. However, in drier climates grape yield might decrease without irrigation and careful vegetation management. Agri‐environmental policies should therefore focus on granting subsidies for the establishment of locally adapted diverse vegetation cover in vineyard inter‐rows. Future studies should focus on analysing the combined effects of local vineyard management and landscape composition and advance research in wine‐growing regions in Asia and in the southern hemisphere.

## 1 INTRODUCTION

Over the centuries, human land use has shaped and altered the majority of our planet's landscapes (Foley et al., 2005). As these human‐shaped ecosystems harbour one of the largest parts of terrestrial biodiversity world‐wide, biodiversity conservation efforts should also focus on the identification and conservation of sustainable land use practices (Tscharntke et al., 2012). Across the globe, intensive land use focusing solely on production is a major driver of global change resulting in the decline of biodiversity, ecosystem functioning and multiple ecosystem services (ES) in agricultural ecosystems (Allan et al., 2015; Foley et al., 2005). Therefore, current and future land use practices should be evaluated concerning trade‐offs between food production and the provision of biodiversity and other ES.

The concept of ES was originally developed to illustrate the benefits that natural ecosystems generate for society and to raise awareness for biodiversity and ecosystem conservation (Westman, 1977). The Millennium Ecosystem Assessment (2005) explicitly considered supporting ES as ecosystem functions underlying other ES like provisioning services (products obtained from ecosystems, for example, food, fibre, water), regulating services (benefits obtained from regulation of ecosystem processes, for example, climate regulation, flood regulation, erosion mitigation) and cultural services (non‐material benefits people obtain from ecosystems, for example, recreational, aesthetic and spiritual gains). Despite the increasing research interest in elucidating the relationships between land use, biodiversity and ES, there are few studies actually measuring multiple ES and their responses to different agricultural management intensities (e.g. Björklund, Limburg, & Rydberg, 1999). In addition, only few studies cover different ES and their multifunctionality in vineyard systems (Winkler, Viers, & Nicholas, 2017).

Viticulture is among the oldest and most profitable forms of agriculture, covering about 7.5 million hectares world‐wide (OIV, 2017). Vineyards cover a very broad range of latitudes and edaphoclimatic conditions, from 4° to 51° in the Northern Hemisphere and from 6° until 45° latitude in New Zealand in the Southern Hemisphere. Vineyards could theoretically offer rather attractive and stable habitats for a range of species, especially in inter‐rows covered by diverse plant species, which are favourable for pollinators (Kehinde & Samways, 2014a) and invertebrates that provide pest control services (Shields, Tompkins, Saville, Meurk, & Wratten, 2016). Therefore, vineyards may benefit from and contribute to conservation and ES provision, especially as wine consumers increasingly appreciate environmentally friendly farming practices (Viers et al., 2013). However, vineyards are also among the most intensively managed agroecosystems, typically involving numerous pesticide applications, soil tillage operations and high landscape simplification (Nicholls, Altieri, & Ponti, 2008). The most important groups of pesticides sprayed in vineyards are fungicides, herbicides and to a lesser extent also insecticides. The intensive use of herbicides in vineyards is a global problem for the environment and humans as residues have been found in surface water, groundwater (Louchart, Voltz, Andrieux, & Moussa, 2001), grape juice and wines (Ying & Williams, 1999).

Vineyard management is influenced by climate, irrigation, soil type, grapevine variety, agri‐environmental policies and most importantly winegrowers’ decisions and attitudes. In general, inter‐row vegetation is assumed to be beneficial for erosion prevention and biodiversity provision in vineyards. Nevertheless, inter‐row vegetation is often removed due to perceived competition between it and vines for water and nutrients (Pardini, Faiello, Longhi, Mancuso, & Snowball, 2002). However, not all studies show the expected decline in grape yields (e.g. Ruiz‐Colmenero, Bienes, & Marqués, 2011; Tesic, Keller, & Hutton, 2007), but similar or even higher yields in vineyards with vegetation cover in the inter‐rows (Mercenaro, Nieddu, Pulina, & Porqueddu, 2014; Sweet & Schreiner, 2010). These contrasting results might be explained by climatic differences, the use of irrigation, vegetation type and management, which depicts the necessity of a quantitative review.

Most winegrowers control ground vegetation by means of tilling, mulching or herbicide applications. Intensive tillage has been shown to decrease plant and animal species diversity for some taxa (Kazakou et al., 2016; Paoletti et al., 1998). However, others revealed no significant effects or variable and conflicting responses to herbicide treatments (Caprio, Nervo, Isaia, Allegro, & Rolando, 2015). Besides direct effects on species, vineyard management also affects the provision of certain ES such as grape production, pest control or the prevention of soil erosion (Winkler et al., 2017). Intensive soil tillage and herbicide application trigger soil erosion, which is a threat to biodiversity (Montanarella, 2005) and ES provision (Novara, Gristina, Guaitoli, Santoro, & Cerdà, 2013). Experimental results indicate a severe reduction in erosion rates, when winegrowers use cover crops instead of bare soil management (e.g. Ruiz‐Colmenero et al., 2011). In addition, positive effects of the use of cover crops in vineyard inter‐rows on pest control have been reported (Berndt, Wratten, & Scarratt, 2006; Sanguankeo & León, 2011). However, certain plant species may also increase potential pest species by acting as a host plant (Begum, Gurr, Wratten, Hedberg, & Nicol, 2006), by providing resources or shelter (Danne, Thomson, Sharley, Penfold, & Hoffmann, 2010), or by increasing food web complexity and intraguild predation (Finke & Denno, 2004).

The main objective of this study was to perform a meta‐analysis to identify, whether extensive vineyard vegetation management practices have consequences on biodiversity and associated ES across viticultural regions world‐wide. The supposed trade‐off between provisioning services of wine yield and quality with other ES and biodiversity is of central interest for this study. Therefore, we addressed the following research questions: (1) Does extensive vineyard vegetation management increase biodiversity and ES provision in comparison to conventional practices? (2) Which ES categories or biodiversity parameters respond positively and which respond negatively to extensive vineyard vegetation management? (3) Which environmental parameters alter the response to vineyard management? The outcomes of this study will help to formulate agricultural policy recommendations in order to benefit service‐providing biodiversity and associated ES.

## 2 MATERIALS AND METHODS

### 2.1 Literature search

We conducted a systematic literature search in two major databases, SCOPUS and Web of Science (WoS) Core Collection Database (SCI‐EXPANDED index), for studies that compared ES or biodiversity with different vegetation management (initial database query 25 January 2016; detailed search terms in Appendix S1). This resulted in a total number of 1,429 publications.

After screening those papers by title 489 articles remained and after reading the abstracts for their relevance, 157 articles remained for full‐text screening. Abstract screening was performed by two persons in parallel to cover different fields of expertise and to discuss which articles to include (for the detailed selection process see the PRISMA flow diagram in Figure S1). In the next step articles were screened based on a predefined set of inclusion and exclusion criteria. Only empirical datasets were included that compared at least two different soil or vegetation management treatments. Studies that included (1) less than three spatially independent replicates per treatment level, (2) vineyards under plastic or in greenhouses, and (3) treatments not directly manipulating soil or vegetation management in the vineyards (e.g. application of synthetic or external mulches or the use of different fungicide or insecticide treatments) were excluded. In addition, only studies, which reported means and any dispersion measure of the dependent variable (e.g. *SD* or *SEM*), were used. We contacted the authors of recently published papers with missing data of variance or additional information like irrigation regime of the treatments. Thereof, authors of 11 articles sent adequate datasets for the inclusion in this meta‐analysis. We also screened the reference list of review articles and updated the search on the Web of Science and SCOPUS database on the 20 April 2017, thereby 11 additional articles could be included. In addition, two colleagues provided three datasets from unpublished reports and databases.

### 2.2 Data extraction

In full‐text screening and the follow‐up data extraction the co‐authors participated according to their expertise in viticulture (DPo), pest control (DPa), biodiversity (SK, SW, JZ), microbiology (BL) and hydrology/soil sciences (TB, PS, GG, JG). Each expert needed to document why an article was excluded (most frequently due to missing measures of variation or insufficient spatial replication); and if inclusion criteria were met, data and covariates were collected in a common database. If studies reported the outcome of several different treatments, which differed in species diversity, we only included the treatment with the largest contrast to the control, for example, bare soil vs. cover crop mixtures with highest number of plant species. As an exclusion of those datasets (*n* = 11 studies) did not change overall effect size considerably, this approach did not bias results. In general, we only took the data from the latest year or date if articles presented measurements across multiple time periods or consecutive years, because we expected the largest effect at the end of the study period. If that decision could not be met, we combined these separate effect sizes in one composite effect size measure considering non‐independence of multiple comparisons within a study (Borenstein, Hedges, Higgins, & Rothstein, 2009, formulas 24.1 and 24.2 for two or 24.4 and 24.5 for several outcomes). This process was also used to calculate the combined effect of different localities, taxonomic subgroups within an order or soil layers. The extracted data were double‐checked by the first author for correctness and consistency of terminology. Different measures of variation were converted to standard deviation to enable effect size calculation.

The extracted data were categorized in biodiversity, ES categories (provisioning, regulating and supporting ES) and ES types (Millennium Ecosystem Assessment, 2005, see Table 1 and Table S1). Soil fauna abundance, soil fauna feeding activity, mycorrhiza abundance, microbial biomass and respiration were assigned to the ES nutrient cycling, because these organisms play a key role in litter decomposition and organic matter mineralization in the soil (Wardle et al., 2016). If higher values of effect sizes would mean negative impacts on ES (e.g. abundance of pest species or soil loss), that is, ecosystem disservices, the sign of the effect size was reversed.

**Table 1:** Summary of the ecosystem services (ES) (according to the Millennium Ecosystem Assessment, 2005) and biodiversity datasets extracted from 74 included studies

ES category/biodiversity	ES type/biodiversity	Subset (number of datasets included)	Variable
Biodiversity	Biodiversity	Flora (6)	Plant species richness
		Fauna (18)	Earthworm species richness
			Spider species richness and abundance
			Beetle species richness and abundance
			Grasshopper species richness
			Insect pollinator species richness and abundance (bees, butterflies)
			Bird species richness
Provisioning	Grape quality and quantity	Grape quantity (23)	Grape yield
		Grape quality (22)	Must quality (sugar content, titratable acidity, yeast assimilable nitrogen)
Regulating	Erosion protection	Soil loss (9)	Soil loss
		Erosion‐related soil parameters (8)	Water retention
			Topsoil penetration resistance
			Aggregate stability
			Saturated hydraulic conductivity
	Carbon sequestration	Soil carbon (19)	Soil carbon content
	Pollination	Pollination (2)	Flower visitations
			Seeds per plant
	Pest control	Natural enemy‐related parameters (21)	Abundance of potential natural enemies
			Percentage of parasitism and predation
		Pest‐related parameters (13)	Pest abundance
			Damage per vine and plot
	Soil water balance	Soil water balance (6)	Water stress integral, water loss, volumetric soil water content
Supporting	Soil fertility	Soil biota (17)	Soil fauna abundance (nematodes, earthworms, springtails, Oribatida, invertebrates) and biological quality indicator
			Arbuscular mycorrhiza abundance (fungal spores and colonisation)
		Nutrient cycling processes (17)	Soil fauna feeding activity
			Soil microbial biomass
			Soil microbial respiration and activity
			Soil macronutrient content and availability

### 2.3 Effect size calculation and statistical analyses

We calculated the log‐response ratio (lnR) as the estimate of the effect size because effect sizes are not affected by different variances in the control and treatment groups and results are easily interpretable (Borenstein et al., 2009). Control was defined as high‐intensity inter‐row management (soil tillage or use of herbicides to remove vegetation, conventional or other types of intensive management), whereas treatment was defined as extensive inter‐row vegetation management (vegetation cover, organic or other types of extensive vegetation management). The difference between treatment and control varies from the most extreme studies comparing bare soil with diverse cover crops or natural vegetation in the inter‐rows to studies comparing vineyards using a single species as cover crop in comparison to diverse plant communities. The results are reported as the back‐transformed values of the relative percentage of increase (positive values) or decrease (negative values) in comparison to the control treatment. We chose to analyse data with hierarchical mixed‐effects meta‐analysis models that allow incorporating fixed (moderators), true random effects as well as a nesting factor for effect sizes in the respective sources or articles. As several data points were extracted from a single article, we used the article ID as a nesting factor to avoid violating the assumption that effect sizes are independent from each other. We used the rma.mv function of the metafor package (Viechtbauer, 2010) for r (R Development Core Team, 2017) to fit mixed‐effects models to incorporate the true variation in the effect size variation across studies and the fixed effects by adding moderators (Borenstein et al., 2009). The effects of treatment are significant, if the confidence interval (CI) did not overlap with zero (Borenstein et al., 2009).

We used the following explanatory variables as moderators for the effects of management: (1) irrigation (irrigated or rainfed vineyards); (2) climate according to Köppen–Geiger's classification (Kottek, Grieser, Beck, Rudolf, & Rubel, 2006; Mediterranean, oceanic, steppe and continental climates); (3) study design (single vineyard [block/one vineyard] or several vineyards each with randomized block design [block/several vineyards], or multiple vineyards as replicates); (4) treatment‐control types (bare soil [as a result of tillage, herbicides or both] vs. vegetation cover, conventional vs. organic management or other types of extensive vs. intensive inter‐row vegetation management); (5) vegetation management types (no herbicides vs. herbicide use, no tillage vs. tillage and other types of vegetation management like a combination of herbicides and/or tillage vs. vegetation cover or mulching vs. mowing); (6) ecosystem service category and types according to the Millennium Ecosystem Assessment (2005).

Plots for mean effect sizes and 95% CIs were produced with the r package plotrix (Lemon, 2006). Mixed‐effects models with restricted maximum likelihood estimations for estimating the random effects were selected based on significant *Q*‐statistics for residual heterogeneity of moderators and a model difference in Akaike's Information Criteria for small sample size (AIC_c_) of at least 2 (ΔAIC_c_ >2; cf. Motulsky & Christopoulos, 2003). Models including irrigation had a lower sample size as irrigation data were not available from every study. Therefore, we needed to perform separate mixed‐effects models to compare the respective AIC_c_ values (see Table S2). Multiple comparisons between different moderator levels of mixed‐effects models were performed with the general linear hypotheses (glht) function of the multcomp package (Hothorn, Bretz, & Westfall, 2008).

### 2.4 Publication bias and sensitivity analysis

As studies reporting a significant effect have a higher likelihood of being published than studies with null results, we explored the possibility of publication bias graphically (funnel plot) and statistically (regression test with sample size as predictor; Rothstein, Sutton, & Borenstein, 2005). In addition, we calculated Rosenthal's fail‐safe number (Rosenthal, 1979) to estimate the number of unpublished studies, which would erase the significant effect measured by the meta‐analysis. Furthermore, we calculated hat values as a measure of potential outliers in the space of predictors and standardized residuals to identify influential outliers (Viechtbauer & Cheung, 2010). Effect sizes, which were two times larger than the average hat value and standardized residual values which exceeded 3.0 were considered outliers (Habeck & Schultz, 2015) .

## 3 RESULTS

**Figure 1:**
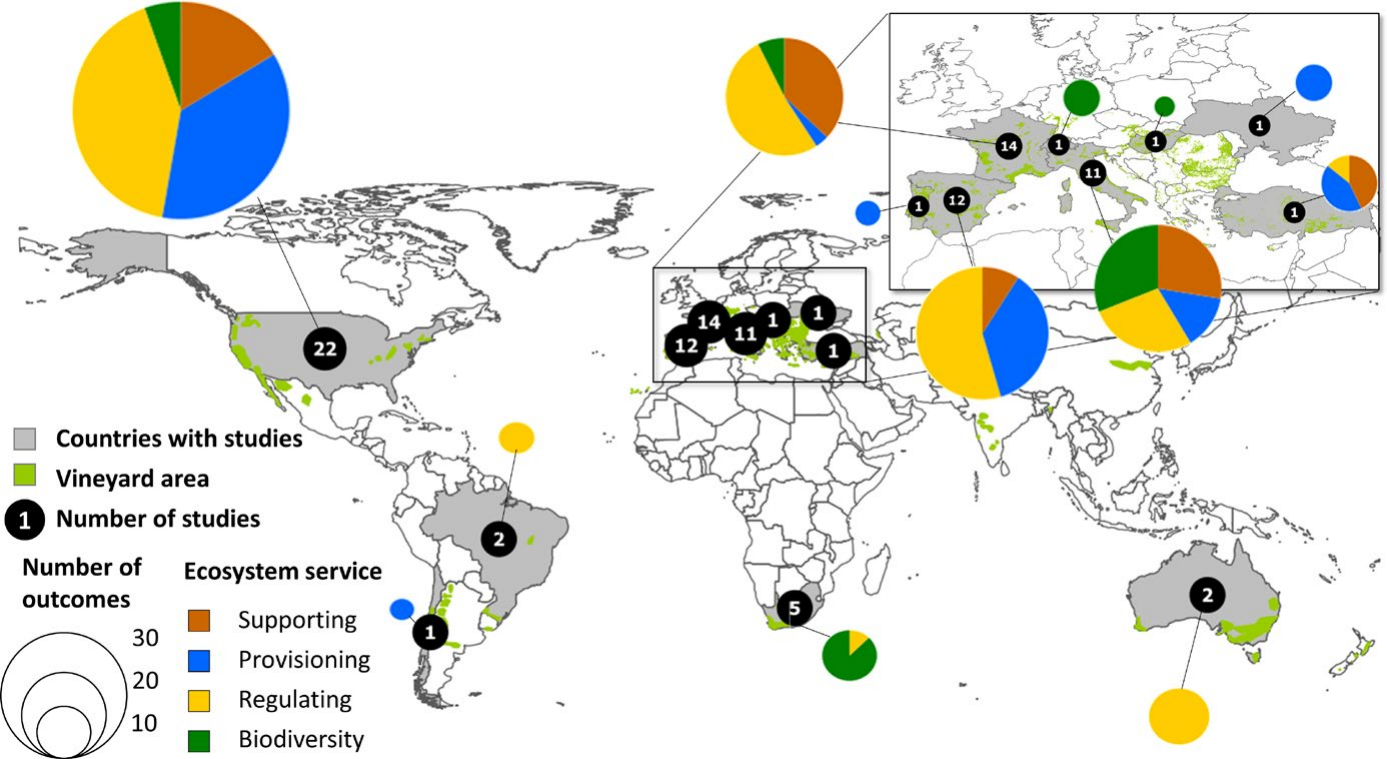
Political map of the world showing the number of involved studies per country and the wine‐growing regions in green shading, number of outcomes symbolize the sample size per country (source: Corine Land Cover for European vineyard area; world‐wide vineyard area based on national maps)

In total, we extracted 181 datasets from 74 articles covering major wine producing regions world‐wide except Asian countries, New Zealand and Argentina (Figure 1, Table S1). The publication dates span from 1992 to 2017. Therefrom, 60 articles originated from the initial search and 14 additional sources from unpublished datasets provided by research colleagues and additional articles from an updated search (see previous chapter and Figure S1).

The different categories of ES were well represented in the datasets with a focus on regulating ES (Figure 1). About 40% of all datasets originated from irrigated vineyards, 50% were rainfed vineyards and the other studies did not provide information on the use of irrigation (Table S1). Most datasets came from vineyards under Mediterranean climates (*n* = 100), oceanic climates (*n* = 56), and steppe or continental climates (*n* = 22; three studies included vineyards from different climates). Most studies implemented randomized block designs within one experimental vineyard (*n* = 113), only few studies implemented block designs in several vineyards (*n* = 12), whereas 56 datasets used individual vineyards as replicate. The majority of studies investigated the effects of bare soil management (mostly due to tillage, sometimes by use of herbicides or both) compared to cover crops or natural vegetation (*n* = 137 datasets). We investigated the effects of conventional vs. organic management in 27 studies and 17 datasets originated from other types of intensive vs. extensive vegetation management like the contrast of single to diverse cover crop species in inter‐rows or mulching vs. mowing of vegetation.

**Figure 2:**
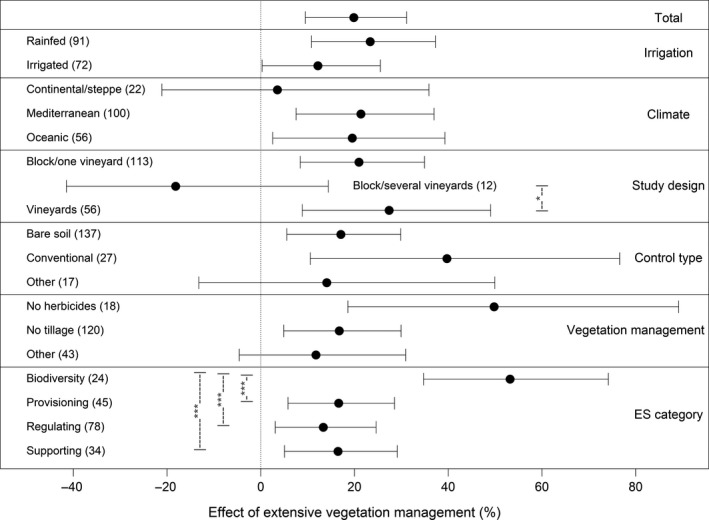
Effects of extensive vegetation management in vineyard inter‐rows on overall effect size. Significant differences between moderator levels are indicated by whiskers with the associated level of significance (*p < .05, ***p < .001). Numbers in brackets show the sample size of the datasets

Overall, there was a 19·8% increase in biodiversity and ecosystem service provision due to extensive vegetation management in comparison to the control treatment (Figure 2). With respect to climate, the effect of extensive vegetation management was significantly positive in studies conducted under Mediterranean and oceanic climate, but not in steppe or continental climates. The mixed‐effects model showed a significant effect of study design on ES and biodiversity (Table S2, Figure 2). The difference between effect sizes of studies using between vineyard replication vs. using within and between vineyard replication (block/several vineyards) was significant, only the latter did not show a positive response to extensive vegetation management (*n *= 12). Studies comparing vegetation cover vs. bare soil (*M *= 17.1%) and organic vs. conventional management (*M *= 39.7%) showed significant positive effects to extensive vegetation management, whereas other studies with less pronounced differences between treatment and control did not. Studies comparing vegetated inter‐rows to herbicide application in inter‐rows resulted in the highest positive effect, followed by the majority of studies investigating tillage vs. vegetation cover. Other forms of vegetation management like the combined use of herbicides and tillage or mulching as control in comparison to more extensive types of vegetation management did not result in an overall significant positive effect.

**Figure 3:**
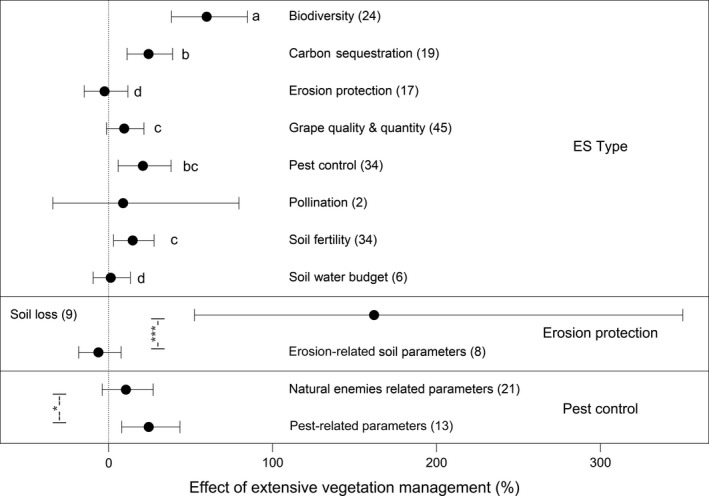
Mean and 95% confidence intervals of the effects of extensive vegetation management in vineyards on biodiversity and ecosystem services (ES) types. Significant pairwise differences between groups are indicated by different letter combinations or by whiskers with the associated level of significance (*p < .05, ***p < .001) for the subsets. Due to the small sample size, pollination was excluded from the pairwise comparisons. Erosion protection and pest control were further split up because subsets (see Table 1) differed significantly from each other in their overall effect sizes. Numbers in brackets show the sample size

The largest mean effect size (*M* = 53.2%) was observed for biodiversity which was also significantly higher than the other ecosystem service categories. However, all ES were significantly positively affected by extensive vegetation management and the inclusion of that moderator significantly improved the model AIC_c_ values (Table S2). The integration of the moderator ecosystem service type improved model fit (alias ΔAIC_c_) more effectively than ES categories (Table S2, Figure 3).

Considering the type of ES in the model, biodiversity benefitted most from extensive vegetation management with a significant difference to all other ecosystem service types. Furthermore, carbon sequestration, pest control and soil fertility showed significant positive responses to extensive vegetation management in the mixed‐effect model with the moderator ES type. If soil erosion was split up into two subsets of parameters measuring soil loss and in general erosion‐related soil parameters, there was a strong positive effect of extensive vegetation management on soil loss mitigation (*M* = 161.9%). This means that soil loss was strongly reduced by using cover crops instead of bare soil management. Pest‐related parameters (positive values show mean lower values of pest species in the treatment), one of the two subsets of the ES‐type pest control, also showed a significant positive response to extensive vegetation management in comparison to the non‐significant effect on natural enemies.

Funnel plots, regressions tests (*z* = 1.79, *p *= .07), and a fail‐safe number of 29,663 showed no sign of publication bias in the presented meta‐analyses (details in Appendix S3).

## 4 DISCUSSION

To our knowledge, this is the first meta‐analysis summarizing the effects of vineyard management on biodiversity and associated ES across the globe. Across studies, extensive vegetation management resulted in a 20% increased biodiversity and ES provision. Irrigation, study design, treatment‐control type, ES category/biodiversity and ES type consecutively improved the model fit. We detected the strongest increase of 50% in biodiversity due to extensive vegetation management. Additionally, carbon sequestration, pest control and soil fertility also showed significant positive responses to extensive vegetation management. A subset analysis of the ES type erosion protection resulted in the largest increase (160%) for studies investigating actual soil loss of vineyards with vegetation cover vs. bare soil management.

Interestingly, irrigation did not increase the positive effect of extensive vegetation management. In fact, rainfed vineyards showed a comparatively larger positive response. The decreased effect in irrigated vineyards might be due to decreasing pest control ES as several studies (Costello, 2008; Irvin, Bistline‐East, & Hoddle, 2016) showed that irrigation may increase the incidence of certain leafhopper pest species as they prefer vigorously growing vines. Such side effects can occur under dry climate conditions, where irrigation is more common and natural enemies cannot control pests (Tscharntke et al., 2016). Climatic effects on the outcome of extensive vegetation management were smaller than expected. In contrast to continental and steppe climates, studies conducted in Mediterranean and oceanic climates showed significant positive responses. Differences were not related to the use of irrigation, as approximately half of all datasets originated from irrigated Mediterranean vineyards, whereas 83% of all datasets in continental or steppe climates descended from irrigated vineyards. Steppe or semi‐arid climates are characterized by rainfall deficiency (Kottek et al., 2006), which increases the need for irrigation.

Previous narrative reviews also found overall positive effects of environmentally friendly management on biodiversity and ES provision of inter‐row vegetation management in vineyards (Guerra & Steenwerth, 2012) and of cover crops in vineyards and olive groves (Pardini et al., 2002). However, some studies indicated trade‐offs between production and other ES (e.g. Morlat & Jacquet, 2003). The review of Guerra and Steenwerth (2012) discussed the relationship of (potential) water stress created by cover crops and concluded that the combination of factors like water regime, cover crop species, management, duration of cover crop establishment, age of vines is very complex and therefore studies show conflicting results. Despite the potential reduction in wine yield and available soil water, water competition between vines and cover crops also creates benefits from some winegrowers (Guerra & Steenwerth, 2012). The reduced vine growth may decrease the costs associated with vineyard operations like fruit thinning and leaf pulling for producing high‐quality wine (Guerra & Steenwerth, 2012). In this meta‐analysis, we could not detect any overall negative effect of inter‐row vegetation cover on grape quantity or quality; nevertheless, in vineyards of dry climates without irrigation grape yields could decrease if vegetation is not carefully managed.

We found a significant difference in effect size dependent on the study design of the considered studies. The non‐significant response of datasets from randomized block designs in several vineyards is most likely the result of the low number of studies, which mainly cover soil loss and grape yield. The type of treatment‐control slightly altered the effects of extensive vegetation management. However, it should be remarked that also sample sizes differed considerably with 56% of all datasets from studies comparing organic vs. conventional management investigated biodiversity. These differences might have increased the associated effect size as biodiversity variables responded strongly positive to extensive vegetation management. Regulations for organic winegrowing do not obligate winegrowers to use cover crops, but in our dataset all organic vineyards used vegetated inter‐rows in the vineyard (only three studies did not include information on inter‐row management). In general, organic management has been shown to increase biodiversity by 30% (Tuck et al., 2014). Inter‐row vegetation management without herbicides was especially beneficial for ES and biodiversity provision. Herbicide application also resulted in the largest negative effect on nematode abundance and soil food web structure compared to tilled or vegetated olive orchards (Sánchez‐Moreno et al., 2015).

Ecosystem services categories and types significantly improved mixed‐effect models and provided insights into possible trade‐offs between biodiversity and ES provision. Overall, extensive vegetation management had an especially large positive effect on biodiversity. This result is very promising, as biodiversity was shown to have positive effects on most ES (Balvanera et al., 2006). Species richness is just one measure of diversity, although the most commonly used and also well acknowledged by the public and policy makers (Batáry, Dicks, Kleijn, & Sutherland, 2015). Hence, further dedicated studies should consider the effects of management intensity on species of conservation concern. Furthermore, increased biodiversity and species abundance might also play a role in sustaining plant–pollinator networks (Kehinde & Samways, 2014a), on which future studies could focus. The few existing literature shows that insect pollinator diversity and abundance is enhanced by organic management (Kehinde & Samways, 2014b) or by reintroducing native plants within and outside vineyards (James, Seymour, Lauby, & Buckley, 2015). This effect is mainly related to a greater number of plant species in vineyard inter‐rows (James et al., 2015; Kehinde & Samways, 2014a) or the availability of more nesting sites for ground nesting species. In addition to local management, the proportion of high‐quality habitats for pollinators at the landscape scale can have strong effects on pollinator diversity and associated ES (e.g. Kennedy et al., 2013). However, this aspect could be not considered in the current study due to a lack of sufficient studies.

Besides biodiversity, all other ES categories showed significant positive responses to extensive vegetation management. However, we could not confirm the supposed trade‐off between provisioning services wine yield/quality vs. biodiversity, regulating or supporting ES. The effect sizes were positive for all ES types but not significant for soil parameters like aggregate stability or saturated hydraulic conductivity, which are assumed to be correlated with a decrease in soil erosion. Obviously, soil loss is a parameter directly addressing erosion, therefore it is most suitable to be used as an indicator for erosion protection despite being highly variable and depending on seasonal conditions (Biddoccu, Ferraris, Opsi, & Cavallo, 2016). In contrast, erosion‐related parameters (aggregate stability, hydraulic conductivity, penetration resistance, porosity, wettability) contain a rather heterogeneous set of indicators that are only indirect measures of soil erosion (Castillo & Gómez, 2016). Positive relationships between aggregate stability and soil water repellency have been reported in a meta‐analysis (Zheng, Morris, Lehmann, & Rillig, 2016). However, as many different aggregate stability indices have been proposed as proxy for soil loss (e.g. Ramos, Nacci, & Pla, 2003), careful consideration is required at indicator selection. The decrease in soil erosion of vegetated inter‐rows is mostly due to the mechanical protection by vegetation and their residues, whereas its impact on other soil physical properties is less intense than the impact on soil erosion, more variable across experiments and so more difficult to detect. Finally, improved soil properties can also enhance carbon sequestration and water filtration (Parras‐Alcántara, Lozano‐García, Keesstra, Cerdà, & Brevik, 2016).

Extensive vegetation management also positively affected soil fertility, which can be attributed to stimulatory effects on soil biota such as earthworms (Briones & Schmidt, 2017). However, we should note that most studies investigating tillage effects on soil biota were conducted in arable crops, but tillage in perennial vineyards is not always detrimental to earthworms (Faber, Wachter, & Zaller, 2017; Vršic, 2011). Besides earthworms, springtails have been studied in vineyard soils. Herbicide‐treated inter‐rows decreased springtail abundance and diversity, whereas tillage reduced only their abundance but not their diversity (Renaud, Poinsot‐Balaguer, Cortet, & Le Petit, 2004). Overall, tillage is known to be an important factor in affecting mycorrhizal communities in soils because it directly affects the integrity of the mycelial network (Verbruggen & Kiers, 2010). Studies on the effects of tillage on mycorrhiza in vineyards are scarce. For example, Trouvelot et al. (2015) found that vegetated inter‐rows favour arbuscular mycorrhizal fungi in the soil and roots of grapevines. Thus, effects of tillage on soil biota will consequently also support ES‐like nutrient cycling and soil formation (Brussaard, de Ruiter, & Brown, 2007). Reduced soil management was also shown to significantly increase carbon sequestration (Zehetner et al., 2015), which links to a wide range of other ES like the contribution to atmospheric CO_2_ regulation (Montanaro, Xiloyannis, Nuzzo, & Dichio, 2017).

Extensive vegetation management also had a significant positive effect on pest control. Taking a closer look, there was a difference between the overall effect size from pest‐related parameters, which showed a significant positive response to extensive vegetation management, whereas natural enemy‐related effect size did not differ significantly from zero. This phenomenon was also detected in other crops such as peach, olive or citrus (Paredes, Cayuela, Gurr, & Campos, 2015). Vegetation cover can increase the diversity and abundance of certain natural enemies that may promote intraguild predation (Finke & Denno, 2004), which in turn can reduce the effectiveness of ground cover for pest control. In addition, some plant species promoted in ground cover can increase, rather than decrease, pest abundance (Danne et al., 2010; Landis, Wratten, & Gurr, 2000). Further research on this ES should be pointed to the analyses of landscape, ground cover composition and trophic relationships between the biodiversity actors.

Most of the studies analysed in this meta‐analysis had an experimental setting in a single vineyard, so it is not possible to analyse the combined effects of local vineyard management and landscape composition. It is crucial to identify the key ecological actors in biological control and their relationships among each other to promote management measures designed for different landscape situations (Straub, Finke, & Snyder, 2008; Tscharntke et al., 2016). It has to be noted, however, that the majority of studies used for the current meta‐analysis were conducted in the USA and Europe, whereas other important wine producing regions such as South America, Australia and New Zealand, or Asia are under‐represented.

Taken together, this meta‐analysis demonstrated that extensive inter‐row vegetation management significantly contributed to the provision of multiple ES and biodiversity conservation in vineyards. As most vineyard vine rows are kept free of vegetation, and vegetation cover is often not maintained year‐round or in every inter‐row, most vineyards contain patches of bare ground. This mosaic of heterogeneous vegetation patches provides beneficial conditions for taxa, which benefit from bare ground like ground‐foraging bird species (Schaub et al., 2010) or wild bees (Potts et al., 2005). Results showed that intensive herbicide use and frequent tillage decreased ES and biodiversity provision. Policy instruments like agri‐environment schemes provide powerful tools, which may change management decisions, as the majority of farmers usually do not consider the effects of management on ES and other externalities. European agri‐environmental policies subsidize farmers to adopt vegetation cover in the vineyard inter‐rows in order to prevent soil erosion. Some schemes encourage farmers not to use herbicides, which were shown to be especially beneficial for ES and biodiversity provision. Despite the overall positive effects of extensive vegetation management, grape quantity and quality may decrease in rainfall‐deficient climates without irrigation (Marques, García‐Muñoz, Muñoz‐Organero, & Bienes, 2010; Ruiz‐Colmenero et al., 2011). Therefore, cover crop management, like the frequency and timing of mulching or tillage and the choice of plant species has to be adapted to the local climate and weather conditions to balance trade‐offs between wine production, biodiversity and ES provision (Guerra & Steenwerth, 2012). Reduced vegetation management intensity will also benefit winegrowers in the long run as a multitude of ES, such as soil erosion mitigation, soil fertility and pest control, improved.

## AUTHORS’ CONTRIBUTIONS

S.W. and P.B. designed the study; all authors helped with data extraction from publications; S.W. collected and prepared data; S.W. and P.B. analysed data; all authors contributed to the writing of the manuscript. All authors gave final approval for publication.

## DATA ACCESSIBILITY

Data available via Zenodo https://doi.org/10.5281/zenodo.1162154 (Winter et al., 2018).
