# Supplementary material for: Effects of vegetation management intensity on biodiversity and ecosystem services in vineyards: A meta‐analysis
Source: J Appl Ecol. 2018 Mar 4;55(5):2484–95. doi: 10.1111/1365-2664.13124 (PMC6099225; doi:10.1111/1365-2664.13124)

**Figure S1.** PRISMA flow diagram of the screening process of the included datasets and the associated symbols of the number of involved people.


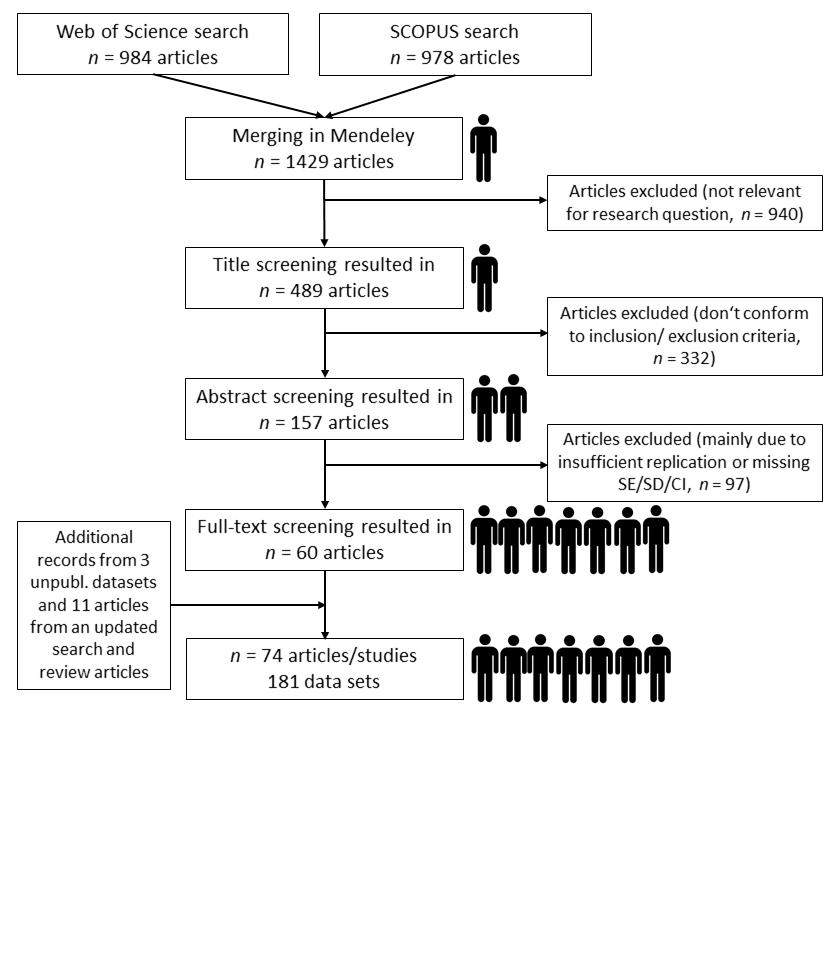

Supplement: Supplementary file 1 [file JPE-55-2484-s001.docx]
