# Supplementary figures and images for: Effects of vegetation management intensity on biodiversity and ecosystem services in vineyards: A meta‐analysis
Source: J Appl Ecol. 2018 Mar 4;55(5):2484–95. doi: 10.1111/1365-2664.13124 (PMC6099225; doi:10.1111/1365-2664.13124)

**Figure S2.** Funnel plot showing the relationship between effect size and sample size of datasets.


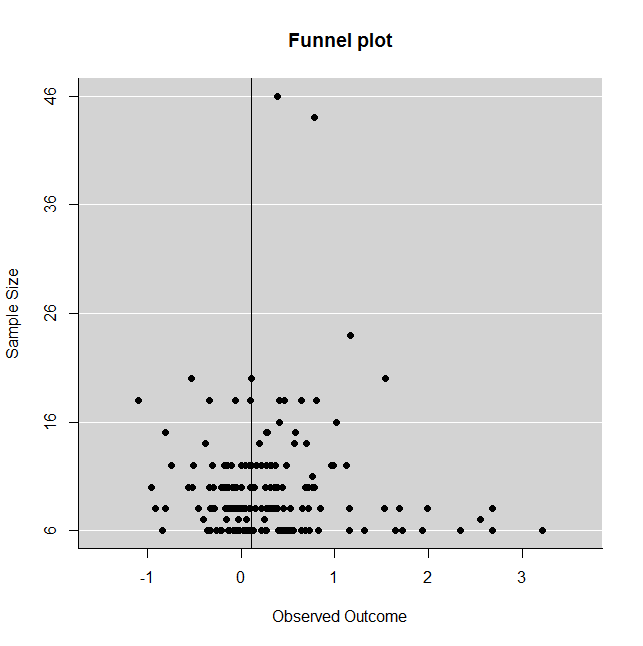

Supplement: Supplementary file 2 [file JPE-55-2484-s002.docx]
