# Supplementary material for: Effects of vegetation management intensity on biodiversity and ecosystem services in vineyards: A meta‐analysis
Source: J Appl Ecol. 2018 Mar 4;55(5):2484–95. doi: 10.1111/1365-2664.13124 (PMC6099225; doi:10.1111/1365-2664.13124)
Supplement: Supplementary file 4 [file JPE-55-2484-s004.pdf]

Table S1. List of 181 datasets from 74 studies included in this meta-analysis. Irrig = irrigation; *N* = sample size; climate type according to Köppen–Geiger"s classification (Kottek, Grieser, Beck, Rudolf, & Rubel, 2006; med = Mediterranean, oceanic, cont-step = continental and steppe climates); study design: block/1 vin = single vineyard with randomized block design, block/sev vin = several vineyards with replicated block or vin = vineyards as replicates; control type: BS = bare soil, CO = conventional management, OT = other type of vegetation management; Veg = vegetation management: TI = tillage, HE = herbicides, OT = other type of vegetation management including combined tillage and herbicide application in inter-rows; NA – missing information; the algebraic sign of log response ratio (lnR) was changed if larger effect sizes caused an overall negative effect on ES provision, e.g. higher soil loss decreases ES provision (lnR*); grey background: we combined effect sizes from different years, localities, taxonomic subgroups within an order or soil layers for that dataset. The source Daane & Costello 1998 includes different study sites, therefore vegetation management differs within the extracted datasets of that study.

**Source**	**Country**	**ES category/ biodiversity**	**ES type/ biodiversity**	**Subset**	**Variable**	**Irrig**	**Climate**	**Study design**	**Control type**	**Veg**	***N***	**lnR***	**var**
Adams, 2011	USA	provisioning	grape quality & quantity	grape quantity	grape yield	yes	med	block/sev vin	BS	HE	12	-0.14165	0.0763
Adams, 2011	USA	regulating	carbon sequestration	soil carbon	soil carbon content	yes	med	block/sev vin	BS	HE	12	0	0.0231
Amaral et al., 2012	Brazil	regulating	carbon sequestration	soil carbon	soil carbon content	no	ozeanic	block/1 vin	BS	OT	12	0.0469	0.0009
Barrio et al., 2011	Spain	provisioning	grape quality & quantity	grape quantity	grape yield	no	med	block/sev vin	BS	TI	6	-0.3263	0.0098
Barrio et al., 2011	Spain	regulating	pest control	pest related param.	damage per vine	no	med	block/sev vin	BS	TI	6	0.27011	0.1086
Barrio et al., 2011	Spain	regulating	pest control	pest related param.	damage per plot	no	med	block/sev vin	BS	TI	6	0.05881	0.3369
Barroso et al., 2016	Portugal	provisioning	grape quality & quantity	grape quantity	grape yield	no	med	block/1 vin	BS	TI	8	-0.45636	0.0166
Barroso et al., 2016	Portugal	provisioning	grape quality & quantity	grape quantity	grape yield	yes	med	block/1 vin	BS	TI	8	-0.32424	0.0097
Bartoli & Dousset, 2011	France	regulating	erosion protection	erosion related soil param.	aggregate stability	no	ozeanic	block/1 vin	BS	TI	8	0.04185	0.0023
Bartoli & Dousset, 2011	France	regulating	erosion protection	erosion related soil param.	aggregate stability	no	ozeanic	block/1 vin	BS	TI	8	0.09641	0.0006
Bartoli & Dousset, 2011	France	regulating	carbon sequestration	soil carbon	soil carbon content	no	ozeanic	block/1 vin	BS	TI	8	0.84627	0.0228
Baumgartner et al., 2005	USA	supporting	soil fertility	soil biota	arbuscular mycorrhiza abundance	yes	med	block/1 vin	BS	TI	18	0.46705	0.0596
Baumgartner et al., 2005	USA	supporting	soil fertility	soil biota	arbuscular mycorrhiza abundance	yes	med	block/1 vin	BS	TI	18	0.64806	0.0691
Belmonte et al., 2016	Italy	regulating	erosion protection	erosion related soil param.	water retention	no	med	block/1 vin	BS	TI	6	-0.01135	0.0018
Belmonte et al., 2016	Italy	regulating	carbon sequestration	soil carbon	soil carbon content	no	med	block/1 vin	BS	TI	6	0.09442	0.0063
Blavet et al., 2009	France	regulating	erosion protection	soil loss	soil loss	no	med	block/sev vin	BS	HE	6	0.50484	0.3733

Source	Country	ES category/ biodiversity	ES type/ biodiversity	Subset	Variable	Irrig	Climate	Study design	Control type	Veg	*N*	lnR*	var
Botton et al., 2010	Brazil	regulating	pest control	pest related param.	pest abundance (*Eurhizococcus brasiliensis*)	no	ozeanic	block/1 vin	BS	HE	12	0.9637	0.035
Bouffaud et al., 2016	France	supporting	soil fertility	soil biota	arbuscular mycorrhiza abundance	NA	ozeanic	vin	CO	OT	14	0.19986	0.0479
Bouffaud et al., 2016	France	regulating	carbon sequestration	soil carbon	soil carbon content	NA	ozeanic	vin	CO	OT	14	-0.37555	0.069
Brittain et al., 2010	Italy	biodiversity	biodiversity	fauna	insect pollinator abundance (butterflies, bees)	yes	ozeanic	vin	CO	OT	12	1.12393	0.5005
Brittain et al., 2010	Italy	biodiversity	biodiversity	fauna	insect pollinator morpho species richness (butterflies, bees)	yes	ozeanic	vin	CO	OT	12	0.36772	0.1187
Brittain et al., 2010	Italy	biodiversity	biodiversity	fauna	insect pollinator species richness (bees)	yes	ozeanic	vin	CO	OT	12	0.10725	0.0273
Brittain et al., 2010	Italy	regulating	pollination	pollination	seeds per plant	yes	ozeanic	vin	CO	OT	12	-0.17398	0.0452
Bruggisser et al., 2010	Switzerland	biodiversity	biodiversity	fauna	grasshopper species richness	no	ozeanic	vin	OT	OT	10	-0.52096	0.0616
Bruggisser et al., 2010	Switzerland	biodiversity	biodiversity	fauna	spider species richness	no	ozeanic	vin	OT	OT	10	-0.04915	0.012
Bruggisser et al., 2010	Switzerland	biodiversity	biodiversity	flora	plant species richness	no	ozeanic	vin	OT	OT	10	0.25644	0.11
Burns et al., 2015	USA	regulating	carbon sequestration	soil carbon	soil carbon content	NA	med	vin	BS	TI	14	0.57521	0.161
Caprio et al., 2015	Italy	biodiversity	biodiversity	fauna	beetle abundance (carabids)	no	ozeanic	vin	BS	HE	12	-0.51019	0.1982
Caprio et al., 2015	Italy	biodiversity	biodiversity	fauna	spider abundance	no	ozeanic	vin	BS	HE	12	0.32686	0.0428
Celette et al., 2005	France	regulating	erosion protection	erosion related soil param.	saturated hydraulic conductivity	no	med	block/1 vin	BS	HE	8	-0.91629	4.7181
Cluzeau et al., 2013	France	biodiversity	biodiversity	fauna	earthworm species richness	no	ozeanic	vin	BS	HE	12	0.11665	0.0542
Cluzeau et al., 2013	France	supporting	soil fertility	soil biota	Soil fauna abundance (earthworms)	no	ozeanic	vin	BS	HE	12	-0.105	0.0653
Coll et al., 2011	France	supporting	soil fertility	nutrient cycling processes	soil microbial biomass (C)	no	med	vin	CO	TI	15	0.27132	0.0094
Coll et al., 2011	France	supporting	soil fertility	soil biota	Soil fauna abundance (earthworms)	no	med	vin	CO	TI	15	-0.80178	0.0888
Coll et al., 2011	France	supporting	soil fertility	soil biota	Soil fauna abundance (nematodes)	no	med	vin	CO	TI	15	0.58016	0.1822
Coll et al., 2011	France	regulating	carbon sequestration	soil carbon	soil carbon content	no	med	vin	CO	TI	15	0.2803	0.0001

Source	Country	ES category/ biodiversity	ES type/ biodiversity	Subset	Variable	Irrig	Climate	Study design	Control type	Veg	*N*	lnR*	var
Coll et al., 2012	France	supporting	soil fertility	soil biota	Soil fauna abundance (nematodes)	no	med	vin	CO	TI	15	0.58016	0.0089
Costello & Danne, 1998	USA	regulating	pest control	natural enemies related param.	abundance potential natural enemies (spider)	yes	med	block/1 vin	BS	OT	10	0.10551	0.0133
Costello & Danne, 2003	USA	biodiversity	biodiversity	fauna	spider species richness	yes	cont-step	block/1 vin	BS	TI	12	0.08961	0.0018
Costello & Danne, 2003	USA	regulating	pest control	natural enemies related param.	percentage of parasitism	yes	cont-step	block/1 vin	BS	TI	12	-0.29952	0.0024
Costello & Danne, 2003	USA	regulating	pest control	pest related param.	pest abundance (leafhopper)	yes	cont-step	block/1 vin	BS	TI	12	0.16038	0.0156
Costello, 2010a	USA	regulating	soil water balance	soil water balance	volumetric soil water content	yes	cont-step	block/1 vin	BS	TI	6	-0.13278	0.0015
Costello, 2010b	USA	provisioning	grape quality & quantity	grape quantity	grape yield	yes	cont-step	block/1 vin	BS	TI	6	-0.12257	0.0174
Daane & Costello, 1998	USA	regulating	pest control	natural enemies related param.	percentage of parasitism	yes	med	block/1 vin	BS	TI	8	0.03131	0.0056
Daane & Costello, 1998	USA	regulating	pest control	pest related param.	pest abundance (leafhopper)	yes	cont-step	block/1 vin	BS	TI	12	-0.7376	0.1669
Daane & Costello, 1998	USA	regulating	pest control	pest related param.	pest abundance (leafhopper)	yes	med	block/1 vin	BS	OT	10	-0.3317	0.0205
Daane & Costello, 1998	USA	regulating	pest control	pest related param.	pest abundance (leafhopper)	yes	med	block/1 vin	BS	TI	8	-0.29239	0.0034
Danne et al., 2010	Australia	regulating	pest control	natural enemies related param.	abundance potential natural enemies (hemiptera)	yes	cont-step	block/1 vin	OT	OT	8	0.154	0.0484
Danne et al., 2010	Australia	regulating	pest control	natural enemies related param.	percentage of predation (of brown apple moths)	yes	cont-step	block/1 vin	OT	OT	8	0.34225	0.0238
Danne et al., 2010	Australia	regulating	pest control	pest related param.	pest abundance (coleoptera, hemiptera)	yes	cont-step	block/1 vin	OT	OT	8	-0.294	0.1182
English-Loeb et al., 2003	USA	regulating	pest control	natural enemies related param.	abundance potential natural enemies (Anagrus)	no	cont-step	block/1 vin	OT	OT	8	-0.28146	0.0128
English-Loeb et al., 2003	USA	regulating	pest control	natural enemies related param.	percentage of parasitism (of hemiptera)	no	cont-step	block/1 vin	OT	OT	8	0.28768	0.449
English-Loeb et al., 2003	USA	regulating	pest control	pest related param.	pest abundance (leafhopper)	no	cont-step	block/1 vin	OT	OT	8	0.44849	0.0039
Favretto et al., 1992	Italy	supporting	soil fertility	soil biota	Soil fauna abundance (invertebrates)	no	med	block/1 vin	BS	TI	6	1.31973	0.3228
Favretto et al., 1992	Italy	supporting	soil fertility	soil biota	Soil fauna abundance (Oribatida)	no	med	block/1 vin	BS	TI	6	1.71847	5.6714
Favretto et al., 1992	Italy	supporting	soil fertility	soil biota	Soil fauna abundance (springtails)	no	med	block/1 vin	BS	TI	6	1.93845	3.2902

Source	Country	ES category/ biodiversity	ES type/ biodiversity	Subset	Variable	Irrig	Climate	Study design	Control type	Veg	*N*	lnR*	var
Favretto et al., 1992	Italy	regulating	carbon sequestration	soil carbon	soil carbon content	no	med	block/1 vin	BS	TI	6	0.3973	0.014
Gaigher & Samways, 2014	South Africa	biodiversity	biodiversity	fauna	spider species richness	NA	med	vin	CO	HE	6	0.26622	0.0595
Gaigher & Samways, 2014	South Africa	biodiversity	biodiversity	flora	plant species richness	NA	med	vin	CO	HE	6	0.56004	0.0978
Garcia-Diaz et al., 2017	Spain	regulating	erosion protection	soil loss	soil loss	no	cont-step	block/sev vin	BS	TI	24	1.165	0.7227
Giese et al., 2014	USA	regulating	soil water balance	soil water balance	water loss	no	ozeanic	block/1 vin	BS	HE	12	0.316	0.0017
Gomez et al., 2011	France	regulating	erosion protection	soil loss	soil loss	no	NA	block/sev vin	BS	TI	6	0.82396	1.3752
Hanna et al., 2003	USA	regulating	pest control	natural enemies related param.	abundance potential natural enemies (spider)	yes	cont-step	block/1 vin	BS	TI	6	0.42578	0.1341
Hanna et al., 2003	USA	regulating	pest control	pest related param.	pest abundance (leafhopper)	yes	cont-step	block/1 vin	BS	TI	6	-0.20294	0.1327
Ingels et al., 2005	USA	provisioning	grape quality & quantity	grape quality	sugar content	yes	med	block/1 vin	BS	TI	8	0.00739	0.0004
Ingels et al., 2005	USA	provisioning	grape quality & quantity	grape quality	titratable acidity	yes	med	block/1 vin	BS	TI	8	-0.08536	0.0009
Ingels et al., 2005	USA	provisioning	grape quality & quantity	grape quantity	grape yield	yes	med	block/1 vin	BS	TI	8	-0.1103	0.0739
Ingels et al., 2005	USA	supporting	soil fertility	nutrient cycling processes	soil microbial biomass (PLFA)	yes	med	block/1 vin	BS	TI	8	0.66095	0.0243
Irvin et al., 2016	USA	provisioning	grape quality & quantity	grape quality	sugar content	yes	med	vin	BS	TI	7	-0.02335	0.0005
Irvin et al., 2016	USA	provisioning	grape quality & quantity	grape quantity	grape yield	yes	med	vin	BS	TI	7	0.06313	0.0332
Irvin et al., 2016	USA	regulating	pest control	natural enemies related param.	abundance potential natural enemies (lacewings)	yes	med	vin	BS	TI	7	-0.15341	0.0986
Irvin et al., 2016	USA	regulating	pest control	natural enemies related param.	abundance potential natural enemies (predatory thrips)	yes	med	vin	BS	TI	7	0.24932	0.0387
Irvin et al., 2016	USA	regulating	pest control	natural enemies related param.	abundance potential natural enemies (predators)	yes	med	vin	BS	TI	7	2.55	36.337
Irvin et al., 2016	USA	regulating	pest control	pest related param.	pest abundance (leafhopper)	yes	med	vin	BS	TI	7	-0.40068	0.0351
Isaia et al., 2006	Italy	regulating	pest control	natural enemies related param.	abundance potential natural enemies (spider)	no	ozeanic	vin	BS	OT	14	0.694	0.1853
James et al., 2015	USA	biodiversity	biodiversity	fauna	insect pollinator species richness (butterflies)	yes	NA	vin	BS	HE	8	0.71473	0.1053

**Source**	**Country**	**ES category/ biodiversity**	**ES type/ biodiversity**	**Subset**	**Variable**	**Irrig**	**Climate**	**Study design**	**Control type**	**Veg**	***N***	**lnR***	**var**
James et al., 2015	USA	biodiversity	biodiversity	flora	plant species richness	yes	NA	vin	BS	HE	8	1.68682	0.386
Kehinde & Samways, 2012	South Africa	biodiversity	biodiversity	fauna	insect pollinator species richness (bees)	NA	med	vin	CO	OT	12	-0.1431	0.1295
Kehinde & Samways, 2012	South Africa	biodiversity	biodiversity	fauna	beetle species richness (monkey beetle)	NA	med	vin	CO	OT	12	0.22314	0.3233
Kehinde & Samways, 2014a	South Africa	biodiversity	biodiversity	fauna	insect pollinator species richness	NA	med	vin	CO	OT	10	0	0.0782
Kehinde & Samways, 2014a	South Africa	regulating	pollination	pollination	flower visitations	NA	med	vin	CO	OT	10	0.4454	1.6208
Kehinde & Samways, 2014b	South Africa	biodiversity	biodiversity	flora	flowering plant species richness	NA	med	vin	CO	OT	10	0.10725	0.1963
King & Berry, 2005	USA	supporting	soil fertility	nutrient cycling processes	soil macronutrient content (N)	yes	med	block/1 vin	OT	OT	10	-0.16705	0.0014
Klymenko, 2014	Ukraine	provisioning	grape quality & quantity	grape quality	sugar content	yes	ozeanic	block/1 vin	OT	OT	8	0.01105	0.0013
Klymenko, 2014	Ukraine	provisioning	grape quality & quantity	grape quality	titratable acidity	yes	ozeanic	block/1 vin	OT	OT	8	-0.02532	0.004
Klymenko, 2014	Ukraine	provisioning	grape quality & quantity	grape quantity	grape yield	yes	ozeanic	block/1 vin	OT	OT	8	0.01905	0.0029
Lee & Steenwerth, 2013	USA	provisioning	grape quality & quantity	grape quality	sugar content	yes	med	block/1 vin	BS	TI	6	-0.02151	0.001
Lee & Steenwerth, 2013	USA	provisioning	grape quality & quantity	grape quality	titratable acidity	yes	med	block/1 vin	BS	TI	6	-0.02667	0.0014
Lee & Steenwerth, 2013	USA	provisioning	grape quality & quantity	grape quantity	grape yield	yes	med	block/1 vin	BS	TI	6	-0.08499	0.0031
Marques et al., 2010	Spain	provisioning	grape quality & quantity	grape quality	titratable acidity	no	med	block/1 vin	BS	TI	6	-0.03241	0.0043
Marques et al., 2010	Spain	provisioning	grape quality & quantity	grape quantity	grape yield	no	med	block/1 vin	BS	TI	6	-0.21283	0.0096
Marques et al., 2010	Spain	regulating	carbon sequestration	soil carbon	soil carbon content	no	med	block/1 vin	BS	TI	6	0.06899	0.0015
Marques et al., 2010	Spain	regulating	erosion protection	soil loss	soil loss	no	med	block/1 vin	BS	TI	6	3.21999	137.84
Mercenaro et al.. 2014	Italy	provisioning	grape quality & quantity	grape quality	sugar content	yes	med	block/1 vin	BS	TI	8	0.04674	0.0007
Mercenaro et al.. 2014	Italy	provisioning	grape quality & quantity	grape quality	titratable acidity	yes	med	block/1 vin	BS	TI	8	-0.07551	0.0028
Mercenaro et al.. 2014	Italy	provisioning	grape quality & quantity	grape quantity	grape yield	yes	med	block/1 vin	BS	TI	8	0.2734	0.0185
Morlat & Jacquet. 2003	France	regulating	carbon sequestration	soil carbon	soil carbon content	no	ozeanic	block/1 vin	BS	HE	12	0.31845	3,66E-05
Muscas et al.. 2017	Italy	provisioning	grape quality & quantity	grape quantity	grape yield	yes	med	block/1 vin	BS	TI	8	-0.3083	0.0149

Source	Country	ES category/ biodiversity	ES type/ biodiversity	Subset	Variable	Irrig	Climate	Study design	Control type	Veg	*N*	lnR*	var
Muscas et al.. 2017	Italy	regulating	pest control	pest related param.	pest abundance (mealy bugs)	yes	med	block/1 vin	BS	TI	8	0.08818	0.0009
Nascimbene et al.. 2012	Italy	biodiversity	biodiversity	flora	annual plant species richness	no	ozeanic	vin	CO	OT	18	0.10285	0.0266
Nascimbene et al.. 2012	Italy	biodiversity	biodiversity	flora	perennial plant species richness	no	ozeanic	vin	CO	OT	18	0.40834	0.0243
Novara et al.. 2011	Italy	regulating	erosion protection	soil loss	soil loss	yes	med	block/1 vin	BS	TI	6	0.13118	0.0153
Novara et al.. 2011	Italy	regulating	erosion protection	soil loss	soil loss	yes	med	block/1 vin	BS	TI	6	1.15893	0.0027
Okur et al.. 2015	Turkey	provisioning	grape quality & quantity	grape quality	sugar content	yes	med	block/1 vin	BS	TI	6	0.02208	0.1193
Okur et al.. 2015	Turkey	provisioning	grape quality & quantity	grape quality	titratable acidity	yes	med	block/1 vin	BS	TI	6	-0.02684	0.1102
Okur et al.. 2015	Turkey	provisioning	grape quality & quantity	grape quantity	grape yield	yes	med	block/1 vin	BS	TI	6	-0.35858	0.0606
Okur et al.. 2015	Turkey	supporting	soil fertility	nutrient cycling processes	microbial respiration	yes	med	block/1 vin	BS	TI	6	0.44428	0.0626
Okur et al.. 2015	Turkey	supporting	soil fertility	nutrient cycling processes	soil macronutrient content (N)	yes	med	block/1 vin	BS	TI	6	0.54267	0.1157
Okur et al.. 2015	Turkey	supporting	soil fertility	nutrient cycling processes	soil macronutrient availability (P)	yes	med	block/1 vin	BS	TI	6	0.47853	0.0628
Okur et al., 2015	Turkey	regulating	carbon sequestration	soil carbon	soil carbon content	yes	med	block/1 vin	BS	TI	6	0.69315	0.0066
Ovalle et al.. 2010	Chile	provisioning	grape quality & quantity	grape quantity	grape yield	yes	med	block/1 vin	BS	TI	8	0.39375	0.0063
Paoletti et al.. 1998	Italy	biodiversity	biodiversity	fauna	earthworm species richness	NA	ozeanic	vin	BS	TI	16	1.01983	0.1345
Paoletti et al.. 2015	Italy	biodiversity	biodiversity	fauna	beetle abundance (carabids)	NA	ozeanic	vin	CO	OT	10	-0.18913	0.1525
Paoletti et al.. 2015	Italy	supporting	soil fertility	nutrient cycling processes	soil microbial activity	NA	ozeanic	vin	CO	OT	10	0.3543	0.1267
Paoletti et al.. 2015	Italy	supporting	soil fertility	soil biota	Soil fauna abundance (springtails)	NA	ozeanic	vin	CO	OT	10	-0.14081	0.2729
Paoletti et al.. 2015	Italy	supporting	soil fertility	soil biota	Soil fauna abundance (Oribatida)	NA	ozeanic	vin	CO	OT	10	0.13127	0.1355
Paoletti et al.. 2015	Italy	supporting	soil fertility	soil biota	Soil fauna abundance (earthworms)	NA	ozeanic	vin	CO	OT	10	0.71868	0.2353
Paoletti et al.. 2015	Italy	supporting	soil fertility	soil biota	biological quality indicator	NA	ozeanic	vin	CO	OT	10	0.14027	0.0067
Peregrina et al.. 2012	Spain	regulating	carbon sequestration	soil carbon	soil carbon content	yes	ozeanic	block/1 vin	BS	TI	18	0.8086	0.0002
Pérès et al.. 2008	France	biodiversity	biodiversity	fauna	earthworm species richness	no	ozeanic	vin	BS	HE	8	0	0.0315
Pérès et al.. 2008	France	supporting	soil fertility	soil biota	Soil fauna abundance (earthworms)	no	ozeanic	vin	BS	HE	8	-0.80104	0.0366

Source	Country	ES category/ biodiversity	ES type/ biodiversity	Subset	Variable	Irrig	Climate	Study design	Control type	Veg	*N*	lnR*	var
Pérez -Álvarez et al.. 2013	Spain	provisioning	grape quality & quantity	grape quantity	grape yield	no	ozeanic	block/1 vin	BS	TI	6	0.08701	0.0112
Pérez -Álvarez et al.. 2013	Spain	supporting	soil fertility	nutrient cycling processes	soil macronutrient content (N)	no	ozeanic	block/1 vin	BS	TI	6	-0.83397	0.0162
Pérez -Álvarez et al.. 2015	Spain	provisioning	grape quality & quantity	grape quality	titratable aciditiy	no	ozeanic	block/1 vin	BS	TI	6	0.06585	0.0033
Pérez -Álvarez et al.. 2015	Spain	provisioning	grape quality & quantity	grape quality	sugar content	no	ozeanic	block/1 vin	BS	TI	6	-0.01563	0.0004
Pérez -Álvarez et al.. 2015	Spain	provisioning	grape quality & quantity	grape quantity	grape yield	no	ozeanic	block/1 vin	BS	TI	6	0.09706	0.0205
Pérez -Álvarez et al.. 2015	Spain	supporting	soil fertility	nutrient cycling processes	soil macronutrient content (N)	no	ozeanic	block/1 vin	BS	TI	18	-0.05397	0.0016
Pou et al.. 2011	Spain	provisioning	grape quality & quantity	grape quantity	grape yield	yes	med	block/1 vin	BS	TI	8	-0.12768	0.0858
Pou et al.. 2011	Spain	regulating	carbon sequestration	soil carbon	soil carbon content	yes	med	block/1 vin	BS	TI	8	-0.1466	0.0065
Probst et al.. 2008	France	supporting	soil fertility	nutrient cycling processes	microbial respiration	no	ozeanic	vin	BS	TI	8	0.21468	0.1685
Probst et al.. 2008	France	supporting	soil fertility	nutrient cycling processes	soil microbial biomass (C)	no	ozeanic	vin	BS	TI	8	0.21654	0.1196
Probst et al.. 2008	France	regulating	carbon sequestration	soil carbon	soil carbon content	no	ozeanic	vin	BS	TI	8	-0.03625	0.061
Reeve et al.. 2016	USA	provisioning	grape quality & quantity	grape quality	sugar content	no	med	block/1 vin	BS	TI	10	-0.06424	0.0008
Reeve et al.. 2016	USA	provisioning	grape quality & quantity	grape quality	titratable acidity	no	med	block/1 vin	BS	TI	10	-0.08811	0.0014
Reeve et al.. 2016	USA	provisioning	grape quality & quantity	grape quality	yeast assimilable nitrogen	no	med	block/1 vin	BS	TI	10	-0.95827	0.0258
Reeve et al.. 2016	USA	provisioning	grape quality & quantity	grape quantity	grape yield	no	med	block/1 vin	BS	TI	10	-0.20479	0.0048
Reeve et al.. 2016	USA	regulating	soil water balance	soil water balance	volumetric soil water content	no	med	block/1 vin	BS	TI	10	-0.1338	0.0009
Reinecke et al.. 2008	South Africa	supporting	soil fertility	nutrient cycling processes	soil fauna feeding activity	no	med	block/1 vin	CO	HE	8	1.52819	0.0144
Rodriguez Lovelle et al.. 2000	France	provisioning	grape quality & quantity	grape quantity	grape yield	no	ozeanic	block/sev vin	BS	TI	10	-0.56497	0.0322
Ruiz-Colmenero et al.. 2011	Spain	provisioning	grape quality & quantity	grape quantity	grape yield	no	med	block/sev vin	BS	TI	18	-1.093	0.0354
Ruiz-Colmenero et al.. 2011	Spain	regulating	erosion protection	soil loss	soil loss	no	med	block/sev vin	BS	TI	6	2.33752	19.962
Ruiz-Colmenero et al.. 2011	Spain	regulating	soil water balance	soil water balance	volumetric soil water content	no	med	block/sev vin	BS	TI	18	-0.333	0.0063
Ruiz-Colmenero et al.. 2013	Spain	regulating	erosion protection	erosion related soil param.	aggregate stability	no	med	block/1 vin	BS	TI	6	0.72951	0.3601

Source	Country	ES category/ biodiversity	ES type/ biodiversity	Subset	Variable	Irrig	Climate	Study design	Control type	Veg	*N*	lnR*	var
Ruiz-Colmenero et al.. 2013	Spain	regulating	carbon sequestration	soil carbon	soil carbon content	no	med	block/1 vin	BS	TI	8	0.38015	0.0087
Ruiz-Colmenero et al.. 2013	Spain	regulating	erosion protection	soil loss	soil loss	no	med	block/1 vin	BS	TI	6	1.64998	5.4201
Ruiz-Colmenero et al.. 2013	Spain	regulating	erosion protection	soil loss	soil loss	no	med	block/1 vin	BS	TI	6	2.68383	7.3597
Rusch et al.. 2017	France	regulating	pest control	natural enemies related param.	percentage of predation (avian predation marks)	no	ozeanic	vin	OT	TI	20	-0.53063	0.0867
Rusch et al.. 2017	France	regulating	pest control	natural enemies related param.	percentage of predation (of grape berry moths)	no	ozeanic	vin	OT	TI	20	0.11702	0.041
Rusch et al.. 2017	France	regulating	pest control	pest related param.	pest abundance (grape berry moth)	no	ozeanic	vin	OT	TI	20	1.5433	0.5211
Salomé et al.. 2016	France	supporting	soil fertility	soil biota	Soil fauna abundance (nematodes)	no	med	vin	BS	TI	11	0.75977	0.0236
Salomé et al.. 2016	France	regulating	carbon sequestration	soil carbon	soil carbon content	no	med	vin	BS	TI	46	0.39177	0.0472
Sharley et al.. 2008	Australia	regulating	pest control	natural enemies related param.	abundance potential natural enemies (coleoptera)	yes	cont-step	block/1 vin	BS	TI	10	-0.29627	8.7978
Sharley et al.. 2008	Australia	regulating	pest control	natural enemies related param.	abundance potential natural enemies (parasitoids)	yes	cont-step	block/1 vin	BS	TI	10	0.25886	0.3266
Sharley et al.. 2008	Australia	regulating	pest control	natural enemies related param.	abundance potential natural enemies (spider)	yes	cont-step	block/1 vin	BS	TI	10	0.31366	10.005
Sharley et al.. 2008	Australia	regulating	pest control	natural enemies related param.	abundance potential natural enemies (dermaptera)	yes	cont-step	block/1 vin	BS	TI	10	0.38396	4.7694
Sharley et al., 2008	Australia	regulating	pest control	natural enemies related param.	abundance potential natural enemies (lithobiida)	yes	cont-step	block/1 vin	BS	TI	10	0.69315	38.32
Sharley et al.. 2008	Australia	regulating	pest control	natural enemies related param.	abundance potential natural enemies (ants)	yes	cont-step	block/1 vin	BS	TI	10	0.76414	26.517
Sharley et al.. 2008	Australia	regulating	pest control	natural enemies related param.	abundance potential natural enemies (trichogrammatidae)	yes	cont-step	block/1 vin	BS	TI	10	0.78846	4.0435
Smith et al.. 2008	USA	supporting	soil fertility	nutrient cycling processes	soil microbial biomass (C)	yes	med	block/1 vin	BS	TI	6	0.64378	0.008
Smith et al.. 2008	USA	supporting	soil fertility	soil biota	arbuscular mycorrhiza abundance	yes	med	block/1 vin	BS	TI	6	0.21424	0.0247
Steenwerth & Belina. 2008	USA	supporting	soil fertility	nutrient cycling processes	microbial respiration	no	med	block/1 vin	BS	TI	12	0.98415	0.0403
Steenwerth & Belina. 2008	USA	supporting	soil fertility	nutrient cycling processes	soil microbial biomass (C)	no	med	block/1 vin	BS	TI	12	0.48053	0.3012

Source	Country	ES category/ biodiversity	ES type/ biodiversity	Subset	Variable	Irrig	Climate	Study design	Control type	Veg	*N*	lnR*	var
Steenwerth & Belina. 2008	USA	supporting	soil fertility	nutrient cycling processes	soil macronutrient content (N)	yes	med	block/1 vin	BS	TI	12	0.10763	0.004
Steenwerth & Belina. 2008	USA	regulating	carbon sequestration	soil carbon	soil carbon content	no	med	block/1 vin	BS	TI	12	0.27472	0.0001
Steenwerth et al.. 2013	USA	provisioning	grape quality & quantity	grape quantity	grape yield	yes	med	vin	BS	TI	16	0.40943	0.0187
Sweet & Schreiner. 2010	USA	provisioning	grape quality & quantity	grape quality	sugar content	no	med	block/1 vin	BS	TI	8	0.00425	0.0002
Sweet & Schreiner. 2010	USA	provisioning	grape quality & quantity	grape quality	titratable acidity	no	med	block/1 vin	BS	TI	8	-0.0735	0.0044
Sweet & Schreiner. 2010	USA	provisioning	grape quality & quantity	grape quality	yeast assimilable nitrogen	no	med	block/1 vin	BS	TI	8	-0.16705	0.0223
Sweet & Schreiner. 2010	USA	provisioning	grape quality & quantity	grape quantity	grape yield	no	med	block/1 vin	BS	TI	8	0.02575	0.0078
Sweet & Schreiner. 2010	USA	regulating	soil water balance	soil water balance	volumetric soil water content	no	med	block/1 vin	BS	TI	8	-0.06328	0.0014
Trigo-Córdoba et al.. 2015	Spain	provisioning	grape quality & quantity	grape quality	sugar content	no	med	block/1 vin	BS	TI	6	-0.01762	0.0009
Trigo-Córdoba et al.. 2015	Spain	provisioning	grape quality & quantity	grape quality	titratable acidity	no	med	block/1 vin	BS	TI	6	-0.04546	0.0279
Trigo-Córdoba et al.. 2015	Spain	provisioning	grape quality & quantity	grape quantity	grape yield	no	med	block/1 vin	BS	TI	6	-0.25932	0.0971
Trigo-Córdoba et al.. 2015	Spain	regulating	soil water balance	soil water balance	water stress integral	no	med	block/1 vin	BS	TI	6	-0.21476	0.001
Verhulst et al.. 2004	Hungary	biodiversity	biodiversity	fauna	bird species richness	NA	ozeanic	vin	OT	OT	44	0.78276	0.0284
Virto et al.. 2012	Spain	regulating	erosion protection	erosion related soil param.	aggregate stability	no	ozeanic	block/1 vin	BS	OT	8	1.98874	2.4608
Virto et al.. 2012	Spain	regulating	erosion protection	erosion related soil param.	topsoil penetration resistance	no	ozeanic	block/1 vin	BS	OT	8	2.68558	22.334
Virto et al.. 2012	Spain	regulating	erosion protection	erosion related soil param.	water retention	no	ozeanic	block/1 vin	BS	OT	8	0.30713	0.0035
Virto et al.. 2012	Spain	supporting	soil fertility	nutrient cycling processes	soil microbial biomass (C)	no	ozeanic	block/1 vin	BS	OT	8	0.52798	0.0009
Virto et al.. 2012	Spain	regulating	carbon sequestration	soil carbon	soil carbon content	no	ozeanic	block/1 vin	BS	OT	8	0.31197	0.0134
Virto et al., 2012	Spain	regulating	carbon sequestration	soil carbon	soil carbon content	no	ozeanic	block/1 vin	BS	OT	8	1.15315	1.1917
