# Supplementary material for: Effects of vegetation management intensity on biodiversity and ecosystem services in vineyards: A meta‐analysis
Source: J Appl Ecol. 2018 Mar 4;55(5):2484–95. doi: 10.1111/1365-2664.13124 (PMC6099225; doi:10.1111/1365-2664.13124)
Supplement: Supplementary file 5 [file JPE-55-2484-s005.pdf]

Table S2. Summary table of the tests of moderators, associated heterogeneities (Q) and AICc values for each model without intercept, which decreased AICc values relative to the null model (climate did not improve model fit, therefore not shown; for ecosystem service type and categories see Table 1). As models including irrigation had a lower overall sample size, a separate mixed-effects models is presented.

Dataset	Moderators/Residuals	d.f.	Q	*P*	AICc
Full dataset (n = 181)	Null model (no moderator)	180	6085·80	<0·0001	483·49
	**Vegetation management**	3	20·28	0.0001	482·36
	Residual	178	5644·83	<0·0001	
	**Study design**	3	22·09	<0·0001	480·65
	Residual	178	5967·24	<0·0001	
	**Ecosystem Service category**	4	48·51	<0·0001	462·09
	Residual	177	4308·79	<0·0001	
	**Ecosystem Service type**	8	86·67	<0·0001	**438·31**
	Residual	173	3271·56	<0·0001	
Reduced dataset irrigation (n = 163)	Null model (no moderator)	162	6057·19	<0·0001	464·42
	**Vegetation management**	3	16·50	0·0009	463·26
	Residual	160	5615·68	<0·0001	
	**Irrigation**	2	14·81	0·0006	463·49
	Residual	161	6047·82	<0·0001	
	**Study design**	3	17·14	0·0007	462·30
	Residual	160	5938·66	<0·0001	
	**Treatment-Control type**	3	18·66	0·0003	461·01
	Residual	160	5875·15	<0·0001	
	**Ecosystem Service category**	4	45·09	<0·0001	442·96
	Residual	159	4273·33	<0·0001	
	**Ecosystem Service type**	8	84·85	<0·0001	**418·17**
	Residual	155	3234·99	<0·0001	
