# Supplementary material for: Effects of vegetation management intensity on biodiversity and ecosystem services in vineyards: A meta‐analysis
Source: J Appl Ecol. 2018 Mar 4;55(5):2484–95. doi: 10.1111/1365-2664.13124 (PMC6099225; doi:10.1111/1365-2664.13124)
Supplement: Supplementary file 6 [file JPE-55-2484-s006.pdf]

Supplementary material

Appendix S1. Procedure for literature search in databases, search string and subject area exclusion

The literature search in the databases SCOPUS and Web of Science (WoS) was limited to the timespan 1945-2016 using the topic search (TS). We used the following search terms according to the PICO (Population, Intervention, Comparator and Outcome) elements, which were linked with logical operators in order to include the maximum number of relevant studies covering the effect of vineyard management intensity on biodiversity and ecosystem services:

TS=((vine* OR viticulture OR grape) AND (extensive OR "grass cover" OR "vegetation cover" OR "cover crop" OR mulch* OR organic OR "no spray" OR "no tillage" OR "intensive" OR "bare soil" OR "conventional" OR "sprayed" OR "tilled" OR "soil cultivation") AND ("species richness" OR diversity OR "ecosystem service*" OR pollination OR "biological control" OR predation OR erosion OR "carbon storage" OR "water storage capacity" OR "surface runoff" OR "field capacity" OR "soil loss" OR "soil resistance" OR "soil stability" OR "soil detachment" OR "soil pore system" OR infiltration OR yield)).

The initial search results of 1049 references were refined by excluding the following irrelevant categories in Web of Science to 984 articles: "energy fuels", "marine freshwater biology", "public environmental occupational health", "nutrition dietetics", "agriculture dairy animal science", "virology", "oceanography", "materials science paper wood", "thermodynamics", "polymer science", "pediatrics", "paleontology", "oncology", "medicine research experimental", "veterinary sciences", "telecommunications", "radiology nuclear medicine medical imaging", "parasitology", "otorhinolaryngology", "mechanics", "limnology", "ethics", "endocrinology metabolism", "critical care medicine" and "chemistry medicinal". In SCOPUS the following subject areas (analogous to categories in WoS) were excluded to refine the initial search results of 1216 to 978 articles: "earth and planetary sciences", "medicine", "engineering", "chemical engineering", "energy", "veterinary", "materials science", "physics and astronomy", "economics, econometrics and finance", "business, managing and accounting", "nursing", "arts and humanities", "health professions" and "neuroscience". These two sets of references were imported in the reference manager software Mendeley to remove duplicates, resulting in 1429 articles (see Fig. S1).
