# Supplementary material for: Effects of vegetation management intensity on biodiversity and ecosystem services in vineyards: A meta‐analysis
Source: J Appl Ecol. 2018 Mar 4;55(5):2484–95. doi: 10.1111/1365-2664.13124 (PMC6099225; doi:10.1111/1365-2664.13124)
Supplement: Supplementary file 7 [file JPE-55-2484-s007.pdf]

Appendix S2. References of studies included in this meta-analysis

Adams, K. E. (2011). *Influence of Vineyard Floor Management Practices on Soil Aggregate Stability , Total Soil Carbon and Grapevine Yield.* Master thesis. California Polytechnic State University. https://doi.org/10.15368/theses.2011.170

Amaral, H. F., Sena, J. O. A., Andrade, D. S., Jácome, A. G., & Caldas, R. G. (2012). Carbon and soil microbial respiration in soil from conventional, organic vineyards and comparison with an adjacent forest. *Semina:Ciencias Agrarias*, *33*, 437–448. http://doi.org/10.5433/1679-0359.2012v33n2p437

Barrio, I. C., Villafuerte, R., & Tortosa, F. S. (2011). Can cover crops reduce rabbit-induced damages in vineyards in southern Spain? *Wildlife Biology*, *18*, 88–96. http://doi.org/10.2981/10-110

Barroso, J. M., Pombeiro, L., & Rato, A. E. (2016). Impacts of crop level, soil and irrigation management in grape berries of cv "Trincadeira" (*Vitis vinifera* L.). *Journal of Wine Research*, *28*, 1–12. http://doi.org/10.1080/09571264.2016.1238350

Bartoli, F., & Dousset, S. (2011). Impact of organic inputs on wettability characteristics and structural stability in silty vineyard topsoil. *European Journal of Soil Science*, *62*, 183–194. http://doi.org/10.1111/j.1365-2389.2010.01337.x

Baumgartner, K., Smith, R. F., & Bettiga, L. (2005). Weed control and cover crop management affect mycorrhizal colonization of grapevine roots and arbuscular mycorrhizal fungal spore populations in a California vineyard. *Mycrorrhiza*, *15*, 111–119. http://doi.org/10.1007/s00572-004-0309-2

Belmonte, S. A., Celi, L., Stanchi, S., Said-Pullicino, D., Zanini, E., & Bonifacio, E. (2016). Effects of permanent grass versus tillage on aggregation and organic matter dynamics in a poorly developed vineyard soil. *Soil Research*, *54*, 797–808. http://doi.org/10.1071/SR15277

Blavet, D., De Noni, G., Le Bissonnais, Y., Leonard, M., Maillo, L., Laurent, J. Y., ... Roose, E. (2009). Effect of land use and management on the early stages of soil water erosion in French Mediterranean vineyards. *Soil and Tillage Research*, *106*, 124-136. https://doi.org/10.1016/j.still.2009.04.010

Botton, M., de Melo, G. W. B., de Oliveira, O. L. P., & Onzi, I. (2010). Effect of cover crops on brazilian ground pearl (Hemiptera: Margarodidae) in vineyards [Efeito da cobertura vegetal sobre a pérola-da-terra (Hemiptera: Margarodidae) na cultura da videira]. *Acta Scientiarum - Agronomy*, *32*, 681–684. http://doi.org/10.4025/actasciagron.v32i4.4773 [in Portuguese]

Bouffaud, M. L., Bernaud, E., Colombet, A., Van Tuinen, D., Wipf, D., & Redecker, D. (2016). Regional-scale analysis of arbuscular mycorrhizal fungi: The case of Burgundy vineyards. *Journal International Des Sciences de La Vigne et Du Vin*, *50*, 1–8. http://doi.org/10.20870/oeno-one.2016.50.1.49

Brittain, C., Bommarco, R., Vighi, M., Settele, J., & Potts, S. G. (2010). Organic farming in isolated landscapes does not benefit flower-visiting insects and pollination. *Biological Conservation*, *143*(8), 1860–1867. http://doi.org/10.1016/j.biocon.2010.04.029

Bruggisser, O. T., Schmidt-Entling, M. H., & Bacher, S. (2010). Effects of vineyard management on biodiversity at three trophic levels. *Biological Conservation*, *143*, 1521–1528. http://doi.org/10.1016/j.biocon.2010.03.034

Burns, K. N., Kluepfel, D. A., Strauss, S. L., Bokulich, N. A., Cantu, D., & Steenwerth, K. L. (2015). Vineyard soil bacterial diversity and composition revealed by 16S rRNA genes: Differentiation by geographic features. *Soil Biology & Biochemistry*, *91*, 232–247. http://doi.org/10.1016/j.soilbio.2015.09.002

Caprio, E., Nervo, B., Isaia, M., Allegro, G., & Rolando, A. (2015). Organic versus conventional systems in viticulture: Comparative effects on spiders and carabids in vineyards and adjacent forests. *Agricultural Systems*, *136*, 61–69. http://doi.org/10.1016/j.agsy.2015.02.009

Celette, F., Wery, J., Chantelot, E., Celette, J., & Gary, C. (2005). Belowground interactions in a vine (*Vitis vinifera* L.)-tall fescue (*Festuca arundinacea* Shreb.) intercropping system: Water relations and growth. *Plant and Soil*, *276*, 205–217. http://doi.org/10.1007/s11104-005-4415-5

Cluzeau, D., Descotes, A., Georget, C., Chaussod, Nouaim-Chaussod, R., Peres, G., … Moncomble, D. (2013) *VitiEcoBioSol. The living soil of the Champagne vineyards* (Numero de contrat Ministère/ADEME: 1060C0090). Rennes, Université de Rennes. [in French]

Coll, P., Le Cadre, E., Blanchart, E., Hinsinger, P., & Villenave, C. (2011). Organic viticulture and soil quality: A long-term study in Southern France. *Applied Soil Ecology*, *50*, 37–44. http://doi.org/10.1016/j.apsoil.2011.07.013

Coll, P., Le Cadre, E., & Villenave, C. (2012). How are nematode communities affected during a conversion from conventional to organic farming in southern French vineyards? *Nematology*, *16*, 665–676. http://doi.org/10.1163/156854112X624195

Costello, M., & Daane, K. (1998). Influence of ground cover on spider populations in a table grape vineyard. *Ecological Entomology*, 23, 33–40. http://doi.org/10.1046/j.1365-2311.1998.00108.x

Costello, M., & Daane, K. (2003). Spider and leafhopper (*Erythroneura* spp.) response to vineyard ground cover. *Environmental Entomology*, 32, 1085-1089. https://doi.org/10.1603/0046-225X-32.5.1085

Costello, M. J. (2010a). Grapevine and Soil Water Relations with Nodding Needlegrass (*Nassella cernua*), a California Native Grass, as a Cover Crop. *HortScience*, *45*, 621–627.

Costello, M. J. (2010b). Growth and yield of cultivated grape with native perennial grasses nodding needlegrass or California barley as cover crops. *HortScience*, *45*, 154–156.

Daane, K. M., & Costello, M. J. (1998). Can cover crops reduce leafhopper abundance in vineyards? *California Agriculture*, *52*, 27–33. http://doi.org/10.3733/ca.v052n05p27

Danne, A., Thomson, L. J., Sharley, D. J., Penfold, C. M., & Hoffmann, A. A. (2010). Effects of Native Grass Cover Crops on Beneficial and Pest Invertebrates in Australian Vineyards. *Environmental Entomology*, *39*, 970–978. http://doi.org/10.1603/EN09144

English-Loeb, G., Rhainds, M., Martinson, T., & Ugine, T. (2003). Influence of flowering cover crops on *Anagrus* parasitoids (Hymenoptera : Mymaridae) and *Erythroneura* leafhoppers (Homoptera : Cicadellidae) in New York vineyards. *Agricultural and Forest Entomology*, *5*, 173–181. http://doi.org/10.1046/j.1461-9563.2003.00179.x

Favretto, M. R., Paoletti, M. G., Caporali, F., Nannipieri, P., Onnis, A., & Tomei, P. E. (1992). Invertebrates and nutrients in a Mediterranean vineyard mulched with subterranean clover (*Trifolium subterraneum* L.). *Biology and Fertility of Soils*, *14*, 151–158. http://doi.org/10.1007/BF00346055

Gaigher, R., & Samways, M. J. (2014). Landscape mosaic attributes for maintaining groundliving spider diversity in a biodiversity hotspot. *Insect Conservation and Diversity*, *7*, 470–479. http://doi.org/10.1111/icad.12070

García-Díaz, A., Bienes, R., Sastre, B., Novara, A., Gristina, L., & Cerdà, A. (2017). Nitrogen losses in vineyards under different types of soil groundcover. A field runoff simulator approach in central Spain. *Agriculture, Ecosystems and Environment*, *236*, 256–267. http://doi.org/10.1016/j.agee.2016.12.013

Giese, G., Velasco-Cruz, C., Roberts, L., Heitman, J., & Wolf, T. K. (2014). Complete vineyard floor cover crops favorably limit grapevine vegetative growth. *Scientia Horticulturae, 170*, 256–266. http://doi.org/10.1016/j.scienta.2014.03.011

Gómez, J. A., Llewellyn, C., Basch, G., Sutton, P. B., Dyson, J. S., & Jones, C. A. (2011). The effects of cover crops and conventional tillage on soil and runoff loss in vineyards and olive groves in several Mediterranean countries. *Soil Use and Management*, *27*, 502–514. http://doi.org/10.1111/j.1475-2743.2011.00367.x

Hanna, R., Zalom, F. G., & Roltsch, W. J. (2003). Relative impact of spider predation and cover crop on population dynamics of Erythroneura variabilis in a raisin grape vineyard. *Entomologia Experimentalis et applicata*, *107*, 177–191. http://doi.org/10.1046/j.1570-7458.2003.00051.x

Ingels, C. A., Scow, K. M., Whisson, D. A., & Drenovsky, R. E. (2005). Effects of cover crops on grapevines, yield, juice composition, soil microbial ecology, and gopher activity. *American Journal of Enology and Viticulture*, *56*, 19–29.

Irvin, N. A., Bistline-East, A., & Hoddle, M. S. (2016). The effect of an irrigated buckwheat cover crop on grape vine productivity, and beneficial insect and grape pest abundance in southern California. *Biological Control*, *93*, 72–83. http://doi.org/10.1016/j.biocontrol.2015.11.009

Isaia, M., Bona, F., & Badino, G (2006). Influence of landscape diversity and agricultural practices on spider assemblage in Italian vineyards of Langa Astigiana (Northwest Italy). *Environmental Entomology*, *35*, 297–307. https://doi.org/10.1603/0046-225X-35.2.297

James, D. G., Seymour, L., Lauby, G., & Buckley, K. (2015). Beauty with benefits: butterfly conservation in Washington State, USA, wine grape vineyards. *Journal of Insect Conservation*, *19*, 341–348. http://doi.org/10.1007/s10841-015-9761-x

Kehinde, T., & Samways, M. (2012). Endemic pollinator response to organic vs. conventional farming and landscape context in the Cape Floristic Region biodiversity hotspot. *Agriculture, Ecosystems & Environment, 146*, 162-167. https://doi.org/10.1016/j.agee.2011.10.020

Kehinde, T., & Samways, M. J. (2014a). Insect-flower interactions: network structure in organic versus conventional vineyards. *Animal Conservation*, *17*, 401–409. http://doi.org/10.1111/acv.12118

Kehinde, T., & Samways, M. J. (2014b). Management defines species turnover of bees and flowering plants in vineyards. *Agricultural and Forest Entomology, 16*, 95–101. http://doi.org/10.1111/afe.12038

King, A. P., & Berry, A. M. (2005). Vineyard δ15N, nitrogen and water status in perennial clover and bunch grass cover crop systems of California"s central valley. *Agriculture, Ecosystems and Environment*, *109*, 262–272. http://doi.org/10.1016/j.agee.2005.03.002

Klymenko, N. (2014). Sodding between rows and microbial preparations in the environmentally secure production of grapes. *Acta Horticulturae*, *1032*, 133–138. http://doi.org/10.17660/ActaHortic.2014.1032.17

Lee, J., & Steenwerth, K. L. (2013). "Cabernet Sauvignon" grape anthocyanin increased by soil conservation practices. *Scientia Horticulturae*, *159*, 128–133. http://doi.org/10.1016/j.scienta.2013.05.025

Marques, M. J., García-Muñoz, S., Muñoz-Organero, G., & Bienes, R. (2010). Soil conservation beneath grass cover in hillside vineyards under Mediterranean climatic conditions (Madrid, Spain). *Land Degradation & Development, 21*, 122-131. http://doi.org/10.1002/ldr.915

Mercenaro, L., Nieddu, G., Pulina, P., & Porqueddu, C. (2014). Sustainable management of an intercropped Mediterranean vineyard. *Agriculture, Ecosystems and Environment*, *192*, 95–104. http://doi.org/10.1016/j.agee.2014.04.005

Morlat, R., & Jacquet, A. (2003). Grapevine root system and soil characteristics in a vineyard maintained long-term with or without interrow sward. *American Journal of Enology and Viticulture, 54*, 1–7.

Muscas, E., Cocco, A., Mercenaro, L., Cabras, M., Lentini, A., Porqueddu, C., & Nieddu, G. (2017). Effects of vineyard floor cover crops on grapevine vigor, yield, and fruit quality, and the development of the vine mealybug under a Mediterranean climate. *Agriculture, Ecosystems and Environment*, *237*, 203–212. http://doi.org/10.1016/j.agee.2016.12.035

Nascimbene, J., Marini, L., & Paoletti, M. G. (2012). Organic Farming Benefits Local Plant Diversity in Vineyard Farms Located in Intensive Agricultural Landscapes. *Environmental Management*, *49*, 1054–1060. http://doi.org/10.1007/s00267-012-9834-5

Novara, A., Gristina, L., Saladino, S. S., Santoro, A., & Cerda, A. (2011). Soil erosion assessment on tillage and alternative soil managements in a Sicilian vineyard. *Soil & Tillage Research*, *117*, 140–147. http://doi.org/10.1016/j.still.2011.09.007

Okur, N., Kayikcioglu, H. H., Ates, F., & Yagmur, B. (2015). A comparison of soil quality and yield parameters under organic and conventional vineyard systems in Mediterranean conditions (West Turkey). *Biological Agriculture and Horticulture, 32,* 73-84. http://doi.org/10.1080/01448765.2015.1033645

Ovalle, C., del Pozo, A., Peoples, M. B., & Lavín, A. (2010). Estimating the contribution of nitrogen from legume cover crops to the nitrogen nutrition of grapevines using a 15N dilution technique. *Plant and Soil*, *334*, 247–259. http://doi.org/10.1007/s11104-010-0379-1

Paoletti, M. G., Sommaggio, D., Favretto, M. R., Petruzzelli, G., Pezzarossa, B., & Barbafieri, M. (1998). Earthworms as useful bioindicators of agroecosystem sustainability in orchards and vineyards with different inputs. *Applied Soil Ecology*, *10*, 137–150. https://doi.org/10.1016/S0929-1393(98)00036-5

Paoletti, M. G., Squartini, A., Concheri, G., Rumiati, R. I., Mammi, S., Schievano, E., … Gianesini, A. (2015). *VeneTerroir maggior biodiversità per un miglior vino*. (Misura124). Padova: Università degli studi di Padova. [in Italian]

Peregrina, F., Pérez-Álvarez, E., Colina, M., & García-Escudero, E. (2012). Cover crops and tillage influence soil organic matter and nitrogen availability in a semi-arid vineyard. *Archives of Agronomy and Soil Science*, *58*, S95–S102. http://doi.org/http://dx.doi.org/10.1080/03650340.2011.648182

Pérès, G., Cluzeau, D., Mercier, V., & Guernion, M. (2008). *Influence d"entretien du sol sur l"activité biologique lombricienne en vignoble Beaujolais*. [in French]

Pérez-Álvarez, E. P., García-Escudero, E., & Peregrina, F. (2015). Soil nutrient availability under Cover Crops: Effects on vines, must, and wine in a Tempranillo Vineyard. *American Journal of Enology and Viticulture*, *66*, 311–320. http://doi.org/10.5344/ajev.2015.14092

Pérez-Álvarez, E. P., Pérez-Sotés, J. L., García-Escudero, E., & Peregrina, F. (2013). Cover Crop Short-Term Effects on Soil NO3 --N Availability, Nitrogen Nutritional Status, Yield, and Must Quality in a Calcareous Vineyard of the AOC Rioja, Spain. *Communications in Soil Science and Plant Analysis, 44,* 711–721. http://doi.org/10.1080/00103624.2013.748122

Pou, A., Gulías, J., Moreno, M., Tomás, M., Medrano, H., & Cifre, J. (2011). Cover cropping in Vitis vinifera L. cv. Manto Negro vineyards under Mediterranean conditions: Effects on plant vigour, yield and grape quality. *Journal International Des Sciences de La Vigne et Du Vin*, *45*, 223–234. http://doi.org/10.20870/oeno-one.2011.45.4.1501

Probst, B., Schüler, C., & Joergensen, R. (2008). Vineyard soils under organic and conventional management—microbial biomass and activity indices and their relation to soil chemical properties. *Biology and Fertility of Soils*, *44*, 443–450. http://doi.org/10.1007/s00374-007-0225-7

Reeve, A. L., Skinkis, P. A., Vance, A. J., Lee, J., & Tarara, J. M. (2016). Vineyard floor management influences "Pinot noir" vine growth and productivity more than cluster thinning. *HortScience*, *51*, 1233–1244. http://doi.org/10.21273/HORTSC10998-16

Reinecke, A. J., Albertus, R. M. C., Reinecke, S. A., & Larink, O. (2008). The effects of organic and conventional management practices on feeding activity of soil organisms in vineyards. *African Zoology*, *43*, 66–74. http://doi.org/10.3377/1562-7020(2008)43[66:TEOOAC]2.0.CO;2

Rodriguez-Lovelle, B., Soyer, J. P., & Molot, C. (2000b). Nitrogen availability in vineyard soils according to soil management practices. effects on vine. *Acta Horticulturae*, *526*, 277–285. http://doi.org/10.17660/ActaHortic.2000.526.29

Ruiz-Colmenero, M., Bienes, R., Eldridge, D. J., & Marques, M. J. (2013). Vegetation cover reduces erosion and enhances soil organic carbon in a vineyard in the central Spain. *CATENA*, *104*, 153–160. http://doi.org/10.1016/j.catena.2012.11.007

Ruiz-Colmenero, M., Bienes, R., & Marques, M. J. (2011). Soil and water conservation dilemmas associated with the use of green cover in steep vineyards. *Soil & Tillage Research*, *117*, 211–223. http://doi.org/10.1016/j.still.2011.10.004

Rusch, A., Delbac, L., & Thiéry, D. (2017). Grape moth density in Bordeaux vineyards depends on local habitat management despite effects of landscape heterogeneity on their biological control. *Journal of Applied Ecology*. http://doi.org/10.1111/1365-2664.12858

Salomé, C., Coll, P., Lardo, E., Metay, A., Villenave, C., Marsden, C., … & Le Cadre, E. (2016). The soil quality concept as a framework to assess management practices in vulnerable agroecosystems: A case study in Mediterranean vineyards. *Ecological Indicators*, *61*, 456–465. http://doi.org/10.1016/j.ecolind.2015.09.047

Sharley, D. J., Hoffmann, A. A., & Thomson, L. J. (2008). The effects of soil tillage on beneficial invertebrates within the vineyard. *Agricultural and Forest Entomology*, *10*, 233–243. http://doi.org/10.1111/j.1461-9563.2008.00376.x

Smith, R., Bettiga, L., & Cahn, M. (2008). Vineyard floor management affects soil, plant nutrition, and grape yield and quality. *California Agriculture*, *62*, 184–190. http://escholarship.org/uc/item/79k913pk

Steenwerth, K., & Belina, K. M. (2008). Cover crops and cultivation: Impacts on soil N dynamics and microbiological function in a Mediterranean vineyard agroecosystem. *Applied Soil Ecology*, *40*, 370–380. http://doi.org/10.1016/j.apsoil.2008.06.004

Steenwerth, K. L., McElrone, A. J., Calderón-Orellana, A., Hanifin, R. C., Storm, C., Collatz, W., & Manuck, C. (2013). Cover crops and tillage in a mature Merlot vineyard show few effects on grapevines. *American Journal of Enology and Viticulture*, *64*, 515–521. http://doi.org/10.5344/ajev.2013.12119

Sweet, R. M., & Schreiner, R. P. (2010). Alleyway cover crops have little infuence on pinot noir grapevines (Vitis vinifera L.) in two western Oregon vineyards. *American Journal of Enology and Viticulture*, *61*, 240–252.

Trigo-Córdoba, E., Bouzas-Cid, Y., Orriols-Fernández, I., Díaz-Losada, E., & Mirás-Avalos, J. M. (2015). Influence of cover crop treatments on the performance of a vineyard in a humid region. *Spanish Journal of Agricultural Research*, *13*, e0907, 12 p. http://doi.org/10.5424/sjar/2015134-8265

Verhulst, J., Báldi, A., & Kleijn, D. (2004). Relationship between land-use intensity and species richness and abundance of birds in Hungary. *Agriculture, Ecosystems & Environment*, *104*, 465–473. http://doi.org/10.1016/j.agee.2004.01.043

Virto, I., Imaz, M. J., Fernandez-Ugalde, O., Urrutia, I., Enrique, A., & Bescansa, P. (2012). Soil quality evaluation following the implementation of permanent cover crops in semi-arid vineyards. Organic matter, physical and biological soil properties. *Spanish Journal of Agricultural Research*, *10*, 1121–1132. http://doi.org/10.5424/sjar/2012104-613-11
