# Supplementary material for: Effects of vegetation management intensity on biodiversity and ecosystem services in vineyards: A meta‐analysis
Source: J Appl Ecol. 2018 Mar 4;55(5):2484–95. doi: 10.1111/1365-2664.13124 (PMC6099225; doi:10.1111/1365-2664.13124)
Supplement: Supplementary file 8 [file JPE-55-2484-s008.pdf]

Appendix S3. Detailed results of publication bias and data validity.

Neither the regression test ($z = 1{\cdot}79$, $P = 0{\cdot}07$; only implemented for non-nested model), nor the funnel plot (Fig. S2) indicated publication bias in the overall dataset. Publication bias is the results of the preference of the scientific community to publish and accept studies showing higher effect sizes which show significant differences between treatments. Asymmetric funnel plots, significant regression tests or a low Rosenthal's fail safe number indicate that the involved studies are a biased sample of all relevant studies. Rosenthal's fail safe number was with N = 29663 much larger than the benchmark value of $5 \times N + 10$ (Rosenthal 1979), therefore the results of this meta-analysis seem to be very robust. The influential case diagnostics of the non-nested overall model identified two outliers, but as their stepwise exclusion did not alter model results decisively, we decided not to exclude any data (figures not shown). It should be noted that the influential case diagnostics included in the metafor package could only be conducted for the non-nested full dataset as the method "influence" is currently not implemented for the function rma.mv. The graphical test for influential outliers using hat values and standardized residual values was also negative (Fig. S3).
